# On the climate benefit of a coal-to-gas shift in Germany’s electric power sector

**DOI:** 10.1038/s41598-021-90839-7

**Published:** 2021-06-01

**Authors:** Stefan Ladage, Martin Blumenberg, Dieter Franke, Andreas Bahr, Rüdiger Lutz, Sandro Schmidt

**Affiliations:** Federal Institute for Geosciences and Natural Resources (BGR) , Stilleweg 2, 30655 Hannover, Germany

**Keywords:** Climate-change mitigation, Fossil fuels

## Abstract

Methane emissions along the natural gas supply chain are critical for the climate benefit achievable by fuel switching from coal to natural gas in the electric power sector. For Germany, one of the world’s largest primary energy consumers, with a coal and natural gas share in the power sector of 35% and 13%, respectively, we conducted fleet-conversion modelling for reference year 2018, taking domestic and export country specific greenhouse gas (GHG)-emissions in the natural gas and coal supply chains into account. Methane leakage rates below 4.9% (GWP_20_; immediate 4.1%) in the natural gas supply chain lead to overall reduction of CO_2_-equivalent GHG-emissions by fuel switching. Supply chain methane emissions vary significantly for the import countries Russia, Norway and The Netherlands, yet for Germany’s combined natural gas mix lie with << 1% far below specific break-even leakage rates. Supply chain emission scenarios demonstrate that a complete shift to natural gas would emit 30–55% (GWP_20_ and GWP_100_, respectively) less CO_2_-equivalent GHG than from the coal mix. However, further abating methane emissions in the petroleum sector should remain a prime effort, when considering natural gas as bridge fuel on the path to achieve the Paris climate goals.

## Introduction

**Figure 1:**
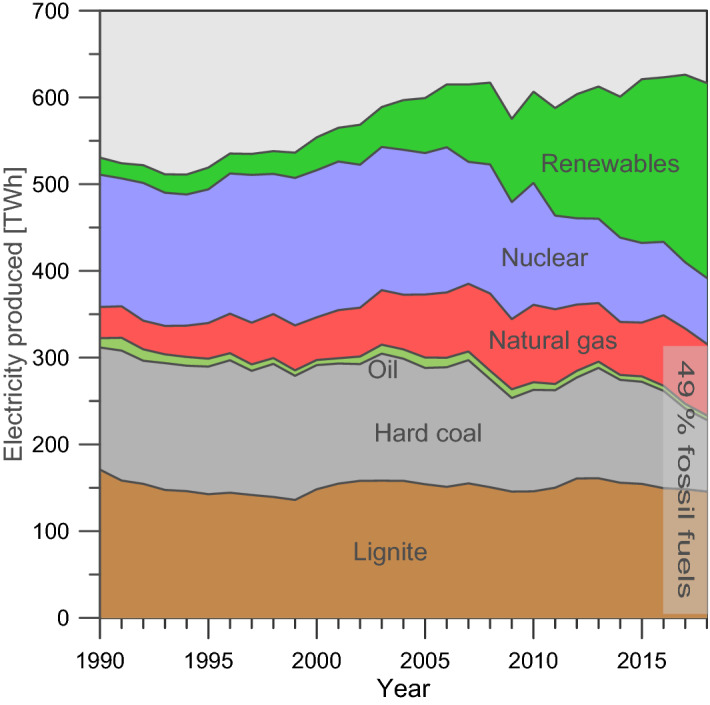
Gross electric power generation in Germany and share of the individual energy sources since 1990. Fossil fuels contribute nearly 50% to the electric power mix in 2018 (data source:AGEB^6^).

Fuel switching from fossil fuels to renewables for electric power generation is a key factor in limiting global warming to 2° or even less to 1.5 °C. To achieve these goals of the 2015 Paris Agreement of the United Nations Framework Convention on Climate Change the European Union has issued the “European Green Deal” agenda, targeting net-zero GHG emissions in the EU member states until 2050. On this path, Germany in particular, as one of the world’s largest consumer of primary energy (3639 TWh in 2018; AGEB^1^, BGR^2^), has set forth plans to successively phase out fossil fuels for electric power generation, which in 2018 had a share of 49% of the generated electric power in Germany (Fig. 1). Hard coal (13%) and lignite (23%) are planned to phase out until 2038 and Germany’s last nuclear power plant will be taken off the grid in 2022 (12%) (Table 1). The reduction in electricity generation by coal and nuclear is supposed to be compensated in large by a conventional mitigation strategy^3^, in particular fuel switching to renewables as well as demand reduction and efficiency gains. Negative emission technologies (NETs), like reforestation or carbon capture and storage and/or utilization (CCS or CCUS)^4^ and upcoming Bioenergy CCS (BECSS) are also considered as well as radiative forcing geoengineering (RFG)^3^. Achieving short-term large-scale GHG reductions, however, remains a considerable challenge, e.g. large-scale onshore CCS/CCUS projects, are not expected to be developed in Germany in the short and medium term, also due to poor social acceptability of these projects.

**Table 1:** German power plant fleet; ^﻿a^reference year 2018; (data sources: AGEB^6^,Wachsmuth et al.^10^).

Fuel	Average power plant efficiency^a^	Fuel share electricity generation^a^	Phase out plan year end	Electric power plant capacity [GW]
Lignite	39%	22.6%	2038	21
Hard coal	44%	12.8%	2038	25
Natural gas	55%	12.8%		22
Nuclear		11.8%	2022	9
Renewables		34.9%		118
Other		5.0%		6

In Germany, and the EU for that matter, it is anticipated, that natural gas remains a key part of the energy system during the net-zero transformation process to cover demand and ensure energy security. Successively, however, natural gas usage might shift from power generation and become part of the building block for hydrogen generation or the production of syngas^5^.

Natural gas power plants produced 13% of the electricity in 2018 in Germany (Table 1). However, they had a nominal utilization of less than 40% in 2018^6^ and thus have a spare capacity. Fuel switching from coal to natural gas cannot be a long-term option to mitigate global warming, since natural gas, completely combusted, still releases about 60% and 50% of CO_2_ emissions compared to hard coal and lignite, respectively^7^. However, on the short term, displacing coal with natural gas in Great Britain has reduced annual national emissions by 6% between 2015 and 2016^8^. Similarly, in the U.S. fuel switching from coal to natural gas and renewables have contributed to a 27% decrease of overall CO_2_-equivalent (CO_2_eq) emissions from electric power generation from 2005 to 2018 and a decrease of 3.7% compared to 1990 emission level^9^. While these reductions were more a side effect of market driven reasons for fuel switching rather than being primarily related to carbon mitigation^8^, these examples demonstrate the impact a short term fuel switch in the electric power sector can have on CO_2_ emission reductions in coal reliant economies, apart from considerably reducing other air pollutants, too.

Methane, the prime constituent of natural gas, however, is a strong GHG in itself, with significantly higher radiative forcing than CO_2_, resulting in high global warming potential (GWP, climate impact relative to carbon dioxide). Over 20 year time span, methane has a high GWP_20_ of ~ 86^11^. Yet, methane is a short-lived climate pollutant (SLCP) with a residence time of approximately 12 years in the atmosphere and the GWP_100_ over a 100 year time span is considerably lower (AR5; 34 and 28, with and without carbon-climate feedbacks, respectively^11^). Thus, methane leakage along the natural gas supply chain may offset climate benefits from fuel switching, especially on short time frames. Alvarez, et al.^12^ analyzed and modeled this effect for the U.S. natural gas network and supply chain and concluded that methane emission rates of natural gas below 3.2% (IPCC, AR4) result in net climate benefits relative to the U.S.-specific coal mix. Using the AR5 updated GWP metrics this break even leakage rate lies at 2.7%^13^.

Methane is especially of concern in the natural gas supply chain. Recent studies, which focused on the oil and gas sector in the U.S.^14,15^ reported significantly higher methane emission rates of the natural gas supply chain than officially reported^9^ and thus challenge methane inventories for the oil and gas sector^16^. The magnitude of overall climate benefits by switching to natural gas instead of coal in terms of reducing GHG for electricity generation is an ongoing scientific debate^17–21^.

Previous studies on a coal-to-gas shift provide a broad range of possible climatic effects^12,18,19,22,23^, and even negative effects are possible^17,24,25^. For instance, an inefficient natural gas sector with considerable losses in the supply chain and an efficient coal sector may counter the advantage of replacing coal with natural gas for power generation^20^. Important, therefore, is to consider country-specific supply and in-use GHG emissions, when appraising any climate benefit by a coal-to-gas shift in the power sector.

With a share of almost 50% electricity generation from fossil fuels (Fig. 1), it is currently debated how quickly Germany can completely convert to a renewable energy supply. With the planned phase-out of coal and nuclear energy, electricity production using natural gas could last longer or even increase its proportions. The objective of this study is therefore to examine whether there is a general reduction potential of overall GHG emissions by fuel switching from coal to natural gas in Germany. Therefore, we model cross-over estimates for a relative climate benefit of using either coal or gas for electricity generation. These results are discussed in the context of the diverse “bottom-up” and “top-down” natural gas leakage studies published to date. “Bottom-up” studies use estimates based on local measurements or emission factors on a component level for broader assessments and “top-down” studies statistically model emissions with regional aircraft or satellite data. We developed several case scenarios for potential leakage rates in the natural gas supply chain for Germany, including a potential LNG supply from the U.S. and examine their GHG reduction potentials compared to coal.

Concerning the terminology, “leakage rate” is used in this study to express natural gas or methane emitted from the natural gas supply chain, including unintended fugitive and intended vented releases, as percentage of the natural gas or methane produced.

## Results and discussion

### Break-even natural gas leakage rates

Based on the specific power generation fuel mix in Germany in 2018, we modelled the natural gas supply chain methane emissions that would lead to a relative climate benefit from a coal to natural gas fuel switch (see section Materials and Methods). This study provides an extensive data compilation comprising the origin, composition, and production conditions of natural gas, lignite and hard coal used for electricity generation in Germany using 2018 as reference year (see supplementary information). Based on this dataset, we adopt the methodology by Alvarez et al.^12^ to derive cross-over estimates for a relative climate benefit of using either coal or gas for electricity generation. Using Monte Carlo simulations, we compute the supply-chain methane leakage rates of natural gas over a 100 year time span, that should not be exceeded to achieve GHG reductions and thus a climate benefit compared to the present fuel mix (break-even leakage rate).

**Figure 2:**
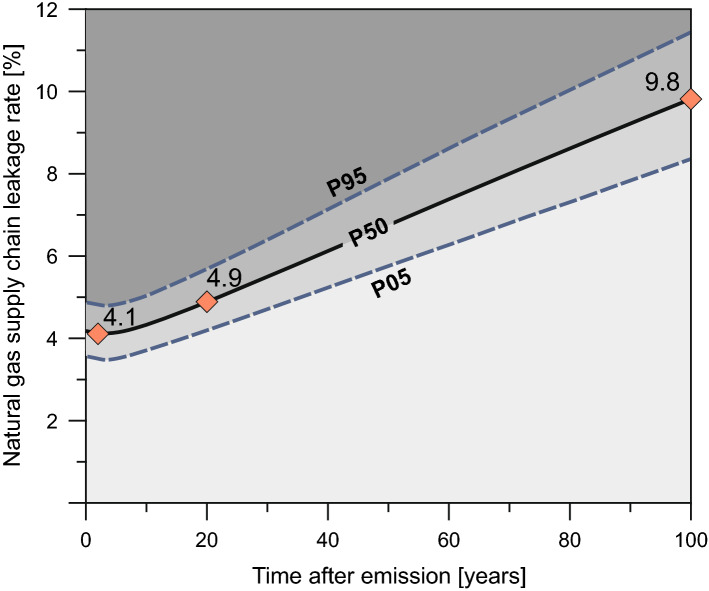
Coal-to-gas fleet conversion model showing the time dependent break-even leakage rates for the natural gas supply chain. Leakage rates below this limit will produce overall less GHGs by natural gas than by coal fired power plants in Germany. To visualize uncertainty, the 5^th^, 50^th^ and 95^th^ percentiles (P05, P50 [median], P95) were calculated for each time step. The medians for GWP_20_, GWP_100_ and an immediate GWP are labeled.

Our model depicts that methane leakage rates below 4.9% (median) on a 20 years time frame (GWP_20_) would lead to less radiative forcing due to natural gas fired power generation than from coal fired. On a 100-years’ time frame (GWP_100_), the leakage rate could even be as high as 9.8% (median). For an immediate climate benefit, the methane leakage rates could be up to 4.1% (median) (Fig. 2). Considering the variance (Table S2) the immediate value shows a range from 3.6 to 4.9 and 4.2 to 5.7 for GWP_20_ and 8.4 to 11.4 for GWP_100_, respectively.

Compared to break-even leakage rates in the U.S. of 2.7%^13^ using IPCC AR5 GWP factors^26^ our modeled immediate break-even leakage rate of 4.1% for Germany is considerably higher. Higher break-even CH_4_ leakage rates for Germany compared to those for the U.S. (and China and India) were also modelled by Tanaka et al.^18^. One explanation for this difference is the higher methane content of hard coals used in Germany compared to the U.S.^12^. However, the main reason lies in the high share of lignite-fired power plants in Germany (64% of total coal in 2018). Lignite has about 30% higher in-use CO_2_ emissions compared to hard coal, taking the energy content and efficiency of the power plants into account (Table 2). These significantly higher emissions from the combustion of lignite are not counterbalanced by the comparatively low methane content and low supply chain CO_2_ emissions due to the domestic origin of lignite. Tanaka et al.^18^, however, modelled higher break-even CH_4_ leakage rates for Germany (9% with GWP_20_; multimetric approach) than we did with our data set (4.9% with GWP_20_). The difference is mainly due to the here determined much lower methane emissions from Germany’s hard coal mix.

**Table 2:** Country specific mean CH_4﻿_ and CO_2_ emissions for power generation in Germany used for fleet-conversion modelling reference year 2018; * see results section; **corrected for power plant fuel efficiency, *** normalized to country specific fuel share; ## Lignite and hard coal combined. See Supplementary Table S1 for full listing.

Fuel	Origin country	Share	Mean emissions [ g/kWh ]				
			Supply chain		In-use	Fuel cycle**	
			CH_4_	CO_2_	CO_2_	CH_4_	CO_2_
**Natural Gas**	Netherlands	22%		3.9	200.8		372.1
	Norway	26%		12.4	202.9		391.5
	Russian Federation	39%		46.1	198.9		445.4
	Germany	8%		18.5	200.1		397.5
	others (av.)	6%		20.2	200.7		401.6
	Gas mix***	100%	*				409.1
**Lignite**	Germany	100%	0.01	10.2	400.1	0.026	1052.1
**Hard coal**	Russia Ferderation	50%	0.88	13.3	349.9	2.0	825.7
	USA	18%	0.33	15.9	325.3	0.8	775.6
	South Africa	3%	0.28	12.6	351.3	0.6	827.2
	Colombia	11%	0.74	10.0	358.9	1.7	838.4
	Poland	1%	1.09	9.6	349.7	2.5	816.5
	Germany	10%	0.64	7.9	331.0	1.4	770.2
	others	9%	0.66	11.6	344.4	1.5	808.9
	Coal Mix***/##	100%				0.6	964.8

### Comparison to reported supply chain methane emissions

A number of GHG life-cycle-analysis (LCA) addressing natural gas usage in the EU have been conducted since 2015^27–29^. These studies focused on a representative group of EU member states, including Germany as a central gas hub in Europe, and specifically analyzed supply chain emissions from the main exporting countries and for relevant import routes via either pipeline transport or LNG. These studies utilized proprietary emission inventory databases (e.g. GaBi^30^; GHGenius LCA database and model) and rely primarily on industry data collections, often on a component-level, and in principle resemble bottom-up inventories.

We compiled the applicable methane emissions data from these LCA studies and compared them with the relevant categories of the UNFCCC National Inventory Reports (NIR) for the Russian Federation, Norway, the Netherlands and Germany. Only very few additional natural gas supply chain methane emission studies have been published to date for these countries. In particular, top down and industry data are not publically available. A listing of the compiled methane emission supply chain literature and data for these countries is given in Supplementary Table S3.

According to this compilation, overall methane leakage rates for natural gas from the Netherlands and Norway, as well as for domestic gas, are in the same order of magnitude of approx. 0.03%. The recent NIR documented slightly higher leakage rates for natural gas, which is produced and transmitted in Germany. Losses during natural gas distribution in the natural gas network appear to be low in Germany and in a similar range^31^. Pipeline gas from the Russian Federation is reported to have an order of magnitude higher leakage rates (details see Supplementary Information). Primarily, this is due to the long distance transcontinental transport from the fields in West Siberia and the Arctic Yamal Peninsula. However, a study focusing on flaring and venting resulted in emission rates of 0.65% (calculated from the implied methane loss rate of 0.43 g/kWh) in the natural gas production sector of Russia in 2012^32^. Nevertheless, all of these “bottom up” inventories are by far below our modeled break-even leakage rates.

**Figure 3:**
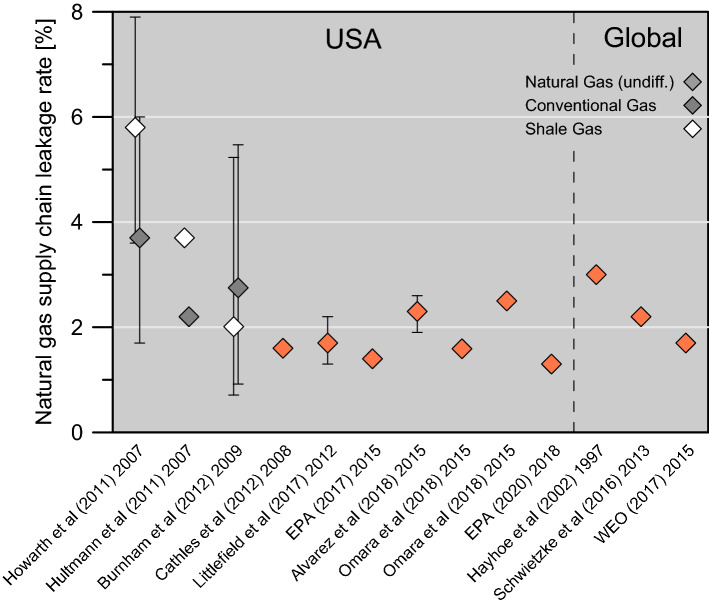
Literature compilation of methane leakage rate assessments with U.S. national and global scope, chronologically ordered. If reported, emissions are differentiated for conventional or shale gas and error bars are given (detailed listing see Supplementary Table S3). Next to the publication year the dataset year is given. Omara et al.^15^ report exclusively on production losses—thus, for comparison, downstream emissions of 1%^14^) were added.

Many “bottom-up” methane inventories, particularly for the Russian upstream oil & gas sector reported to the UNFCCC, appear rather low, compared to recently published studies including, but not limited to, top-down assessments for the U.S. oil & gas sector. For instance, based on the U.S. Greenhouse Gas Reporting Program, the EPA data add up to a nationwide overall methane leakage rate of 1.1% for 2018 (1.3% if also considering methane emissions from the oil sector). We therefore compiled the existing publications of U.S. wide assessments of the last decade, in order to address the possible implications, if theoretically similar methane emission rates would apply for natural gas exported to Germany (Fig. 3 and Supplementary Tables S4 & S5). We also provide a compilation of basin and play wide methane emission studies for the U.S. in the supplement.

Balcombe et al.^33^ have compiled a set of 250 studies and averaged the reported natural gas supply chain leakage rates at 0.97%. The most recent comprehensive studies on methane emissions from the U.S. natural gas supply chain were carried out by Alvarez et al.^14^ and Omara et al.^15^. Alvarez et al.^14^ reported on the complete natural gas supply chain. They estimated a methane loss rate of 2.3% (+ 0.4%/− 0.3%) of the natural gas produced in the U.S in 2015, which is about 60% higher than EPA based leakage rate estimates. The study utilized facility based measurements to assess leakage rates. Most of the methane emissions were found to occur in the production stage.

Omara et al.^15^ focused on methane emissions during production and used a slightly larger dataset of approximately 1000 facility-level measurements. Despite the explicitly stated variability and stochastic nature of the data, according to the authors trends in methane emissions could be derived. Using two different upscaling approaches, they estimated 0.6% and 1.5% leakage rates, respectively, just for the production stage and not the whole supply chain. The upper bound is similar to the leakage rate reported by Alvarez et al.^14^ for this part of the supply-chain. The lower bound is similar to EPA’s estimate^9^. The wide range is due to a few but very large emitters, which are difficult to handle adequately stochastically. Furthermore, considerable differences in emission rates from the various oil and gas provinces in the U.S. exist (Supplementary Table S5). Regions with large emissions show on average leakage rates in the range of 1.6–3.5%, while low emitting provinces were in the range of only 0.031–0.15%^15^. Provinces with a focus on oil production often contribute to high leakage rate assessments for natural gas^34,35^, due to flaring and venting of associated gas, if it cannot be gathered, processed and marketed.

Shale gas production, which is meanwhile contributing more than 60% of dry natural gas production in the U.S.^36^, is another concern for the overall natural gas methane footprint. Simultaneously to the onset of the shale gas boom in the U.S. since 2005 an increase of global atmospheric methane concentrations has been observed^37,38^. Whether coincidence or “cause and effect”, has since been a matter of scientific debate. In a few publications e.g.^25^ very high leakage rates especially for shale gas of 3.6 to 7.9% have been postulated, considerably exceeding any other assessments (Fig: 3). However, other studies contest these estimates^39–41^ and the majority of studies to date do not corroborate a systematically and significantly higher methane footprint of shale gas development and production compared to conventionals (see also Tables S4 & S5)^42–46^. This has recently been further substantiated in a study based on global isotope data and a vast compilation of natural gases from producing fields in the U.S.^47^.

In essence, top-down and facility level U.S. natural gas supply chain leakage rate assessments are often substantially higher than bottom-up (component level) assessments in the U.S. as well as in other countries, albeit they depict high variabilities and statistical uncertainties. Yet, these studies give new insight into potential methane footprints from the natural gas supply chain. Similar investigations for other countries are mostly not available. At present, therefore, it remains ambiguous, whether these predominantly U.S. national investigations on CH_4_ emissions could be representative for other natural gas producing countries. Nevertheless, even then, a coal-to-gas fuel switch in Germany would provide the opportunity for GHG emissions reductions, since nearly all of these studies show supply chain natural gas leakage rates below our median immediate break-even leakage rate of 4.1% for Germany, even more so for GWP_20_ and GWP_100_ scenarios.

Transporting natural gas as liquefied natural gas (LNG) from e.g. the U.S. is an alternative to importing natural gas via pipelines to Germany. We therefore also evaluated the supply chain emissions via LNG from the U.S. (a detailed analysis is given in the Supplementary Table S6). Compared to the pipeline transport from the Netherlands and Norway the natural gas supply chain via LNG from the U.S. exhibits significantly higher GHG emissions from methane loss as well as CO_2_ emissions. However, according to the majority of recent studies, LNG from the U.S. would exhibit only slightly higher methane emissions for the entire supply chain than natural gas from Russia. This is primarily due to the higher reported losses during the production and accumulation of natural gas in the U.S. compared to the significantly lower values reported for the production sector in Russia. However, it has to be considered that in comparison to the U.S., data on emissions in the Russian natural gas supply chain are sparse. We therefore cannot exclude that future studies may alter this general difference between U.S. LNG and Russian pipeline gas. Nevertheless, even the upper limit of the methane emission rates along the U.S. supply chain to Germany/Europe of 2.5% are neither exceeding our modelled median nor the P05 percentile break-even leakage rates on all time scales.

### GHG reduction potential

**Figure 4:**
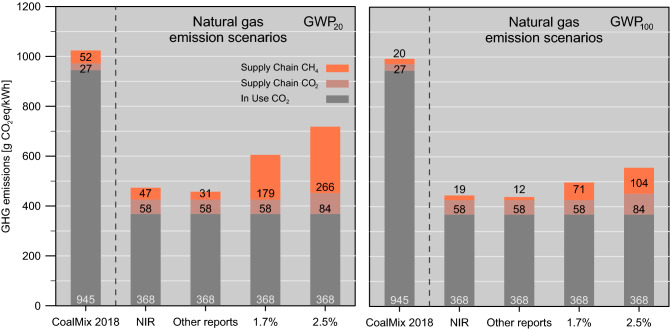
GHG emissions scenarios for Germany compared to 2018 coal mix emissions; left panel using GWP_20_, right GWP_100_. The “NIR scenario” with 0.45% methane leakage rate is based on National Inventory reports and the “Other reports scenario” with 0.32% methane leakage rate from import countries from life-cycle-analysis studies. For comparison, two additional scenarios are presented: a world-wide average “1.7% methane leakage rate scenario” based on IEA, and an upper bound “2.5% scenario”, including e.g. also higher supply-chain CO_2_ emissions from long-distance pipeline or LNG transport.

Absolute GHG emission reductions by a coal-to-gas fuel switch depend on the whole supply chain and in-use CO_2_ and CH_4_ emissions for natural gas from each exporting country. To assess the achievable GHG benefit, we calculated GHG emissions for four different natural gas emission scenarios and compared them to the 2018 coal mix emissions (Fig. 4). We varied the natural gas supply chain methane leakage rates in the different scenarios, while in-use CO_2_ emissions for the natural gas plant fleets remained fixed based on the 2018 gas fuel mix. The first scenario (“NIR scenario”) illustrates potential CO_2_eq emissions using an averaged leakage rate of 0.45% based on the National Inventory Reports. The second scenario “other reports” employs a slightly lower leakage rate of 0.32% founded on other reports, such as LCA studies (see Table S3). The averaged leakage rates for these two scenarios take the import volume fraction from each country for the German gas mix into account. For comparison, two scenarios with higher methane emissions were analyzed. The “1.7% scenario” is based on the global mean 1.7% emission rate assessed by the IEA^52^. The “2.5% scenario” resembles the upper bound based on our compilation of recently published methane emission assessments mostly for the U.S. oil & gas sector. To account for the relatively higher CO_2_ emissions for long-distance pipeline or LNG transport, these CO_2_ emissions were added in the “2.5% scenario” (see Table 2). In particular, the latter scenario includes potentially higher methane emissions from methane slip^7,53,54^.

The resulting CO_2_eq emissions throughout all the natural gas scenarios are considerably lower than for coal (Fig. 4). The largest emission reduction potential of 55% (GWP_20_; 56% with GWP_100_) exists for the “other reports” scenario, having the lowest methane leakage rates in the natural gas supply chain. The “NIR” scenario depicts a similar reduction potential. Even the “2.5% scenario” has ~ 30% and ~ 44% reduced emissions compared to coal, GWP_20_ and GWP_100_ respectively. This is in agreement with Tanaka et al.^18^, who also found a climate benefit from a coal-to-gas switch in Germany using a different methodology and based on a global database.

The absolute numbers presented here, with 2018 as a base, result from an extensive survey of Germany-specific emissions along the natural gas and coal supply chains and in-use GHGs. These values are expected to vary in the future, as there will be changes in the fuel mix for electricity generation in Germany, as well as variations in imports from different countries. However, we consider the estimated break-even levels and the magnitude of supply chain emissions to be robust and covered by the range of the presented scenarios.

In 2018, the total anthropogenic greenhouse gas emissions in Germany accounted for 858 MMtons CO_2_eq based on the older AR4 GWP_100_ metrics^7^. Using this metric here, a coal-to-gas switch in Germany has the potential to reduce the anthropogenic GHG emissions in Germany between 11% and 14%. In contrast, an entire coal-to-renewables switch would result in approx. 24% less GHG emissions, nearly doubling this reduction potential. A complete fossil fuel switch to renewables in the electric power sector could even lead to ~ 30% GHG reductions in Germany. This underpins that natural gas can only be seen as a bridging fuel towards a carbon-free power system in Germany. In addition, it appears crucial that leakage rates in the natural gas supply chain should be further abated by policy regulations and industrial practice (e.g. United Nations Environment Program's Oil and Gas Methane Partnership, OGMP). However, our data demonstrate that on the path to a net-zero GHG energy system a short term coal-to-gas switch of the German power sector has the potential to reduce GHG emissions significantly.

## Materials and methods

### “Fleet conversion” emissions modelling

Comparing GHG emissions of fossil fuels emitting both, CH_4_ and CO_2_ is complex because of the much shorter atmospheric lifetime of CH_4_ relative to CO_2_. To overcome this problem, we adopt the “fleet conversion” modelling method of Alvarez et al.^12^ and compare the cumulative radiative forcing of emissions from using either coal or natural gas for the generation of an equal amount of electric energy over a 100-years’ continuous time span (Eq. 1):

1 $${\mathbf{E}}Gas_{{CH4}} \left[ \% \right] = \left[ {\frac{{{\mathbf{E}}Coal_{{{\text{co}}2}} - {\mathbf{E}}Gas_{{{\text{co}}2}} }}{{{\text{GWP}}({\text{t}})}} + {\mathbf{E}}Coal_{{CH4}} } \right]*\frac{{100}}{{mass_{{\left( {Gas*kWh^{{ - 1}} } \right)}} }}$$

where **E** denotes emissions or leakage (**E***Gas*_*CH4*_ = potential methane leakage of the natural gas supply chain and in-use CH_4_ emissions), and GWP(t) (see Eq. (2)) is the time-dependent global warming potential (GWP) of methane versus CO_2_. Mass methane (CH_4_) used to generate one unit of electric energy considering the power plant efficiency is denoted as mass_(Gas * kWh_^*-1*^_)_.

Global warming potentials (GWPs) were established to allow for comparison among different greenhouse gases at one point in time after emission, e.g. after 100 years, or 20 years. To enable a straightforward comparison of fuel-switch options, Alvarez et al.^12^ suggested that plotting the relative radiative forcing of the individual fuels as a function of time would be more useful than using static GWPs. The time-dependent GWP (Eq. (2); t denotes time) thus is given as

2 $$GWP\left(t\right)= \frac{ {WP(t)}_{CH4}}{{WP(t)}_{CO2}}$$

For a fleet conversion and coal-to-gas shift, assuming constant emissions over time, the time-dependent warming potential WP(t) of either CO_2_ or CH_4_ is given by Alvarez et al.^12^

3 $$WP(t)_{{CH4}} = = GWP^{\prime}\left[ {\tau _{{CH4}} t - \tau _{{CH4}}^{2} (1 - e^{{ - t/\tau _{{CH4}} }} )} \right],$$

4 $$WP(t)_{{CO2}} = a_{0} \frac{{t^{2} }}{2} + \sum\limits_{{i = 1}}^{3} {a_{i} } \left( {\tau _{i} t - \tau _{i}^{2} \left( {1 - e^{{ - {t \mathord{\left/ {\vphantom {t {\tau _{i} }}} \right. \kern-\nulldelimiterspace} {\tau _{i} }}}} } \right)} \right)$$

where ${\tau }_{CH4}$ = 12.4 years (Eqs. 3 & 4), the average time of CH_4_ being stable in the atmosphere^26^, and GWP’ is the global warming potential of CH_4_ relative to CO_2_. Here we use an AR5 value for GWP’ of 120.5, according to the IPCC^11^.

In order to incorporate natural, geological and technological variability of the key input parameters and to quantify uncertainty ranges, Monte Carlo simulations and classical error propagation were conducted.

### Model input data

In the frame of this study, we conducted a literature survey on GHG emission parameters along the natural gas and coal supply chains, as well as for in-use CH_4_ and CO_2_ emissions for power plants in Germany (for 2018). See Supplementary Table S1 for a univariate characterization of the input parameters and parameter distributions (Supplementary Figs. S1 to S5).

### Power plant efficiencies

We here are using average efficiencies as officially reported for Germany of 44% for hard coal, 39% for lignite, and 55% for natural gas fired power plants in year 2018 (Table 1). These efficiencies are calculated using the “Finnish method”, thus differentiating between electricity and heat generation in combined-cycle power plants^7^. A similar mean efficiency of 56% (min 55%, max 58%) for gas power plants and of 37% (min 33%, max 42%) for the coal mix used for power generation in Germany has been employed by Tanaka et al.^18^.

We calculated a mean consumption of 122.3 g CH_4_/kWh generated based on the heating value of the individual gas mix for Germany and taking the power plant efficiency into account. A variance of this value was considered using a minimum of 110 g CH_4_/kWh^18^ and a maximum of 134.6 g CH_4_/kWh, thus giving a symmetrical distribution.

### Coal fuel mix

In 2018, 22.6% of the electricity in Germany was produced from lignite and 12.8% from hard coal^55^, 100% of the lignite and 10% of the hard coal were mined in Germany^56^. About 50% of the hard coal demand for electricity generation were supplied from the Russian Federation, followed by the USA (18%), Colombia (11%), South Africa (3%), and Poland^2^. Following the closure of the last two hard coal mines at the end of 2018, Germany’s hard coal supply now relies entirely on imports.

### Coal supply chain methane emissions

Overviews of methane emissions from coal mining were compiled by Oberschelp et al.^57^ and specifically for Germany by the Federal German Environment Agency (UBA)^58^. Our collation of coal methane content data for coal and lignite used in Germany is mostly based on these studies, with some updated and additional data, i.e. for Colombia and South Africa (Table S1). Mass bulk coal methane contents have been normalized to their specific energy density using reported heating values for the individual coals^59^.

Hard coal mined in Germany had an average methane content of 5 to 10 m^3^ CH_4_/t^60^, corresponding to a mean of 0.64 g CH_4_/kWh (lower than ~ 1.7 g CH_4_/kWh used in another study^57^). For the U.S., 2 m^3^ CH_4_/t has been suggested^12^ corresponding to “low-gassy coals”, which is 75% lower than the “gassy-coal” case. We used this as minimum methane content for the U.S. hard coals and the maximum was set to the average reported by Oberschelp et al.^57^. Methane contents of Russian hard coals are most probably higher than those from U.S. coals. According to the IEA^61^, for instance, Russian coal mines contain on average 11.6 m^3^ CH_4_/t coal. However, this is not representative for steam coal exports from Russia to Germany, which are largely delivered from the Kuznetsk Basin in West-Siberia, the most important Russian hard coal producing basin. The Kuznetsk Basin steam coal production predominantly (77% in 2018) derives from surface mines^2^. These coals have a much lower methane content than coals from deep mines^61^. We therefore accounted for the different Russian coal mine depths on the coal methane contents and employed the lower range of methane content for coals in coal seams from shallow depths (< 300 m) of 2–15 m^3^/t as reported by the IEA^61^. This corresponds to a mean of 8.5 m^3^ CH_4_/t (0.88 g CH_4_/kWh; Table 2), which is considerably less than other estimates (~ 14.2 m^3^ CH_4_/t^57^; 11.6 CH_4 _m^3^/t^61^).

In contrast to hard coal, lignite is thermally less mature and the methane content is therefore much lower. We apply Germany-specific lignite emission values of 0.01 g CH_4_/kWh (upper limit at 0.02 g for Monte Carlo simulations)^2,7,62^.

According to our investigation, coal-based electric power generation resulted in mean methane emissions of 0.6 g CH_4_/kWh (Table 2). This value is based on the country-specific methane content of hard coals imported to Germany, the coal mix and power plant efficiencies. It is considerably lower than the mean value of 5.5 g CH_4_/kWh for Germany from the “ecoinvent” database used by Tanaka et al.^18^.

### Coal supply chain CO_2_ emissions

Varying transport distances are the main drivers for differences in coal supply chain CO_2_ emissions. To estimate country-specific transport emissions, we use average distances from the coal mines to the appropriate export ports taking the varying railroad route conditions (e.g. grade of electrification) into account. This results in relatively low values of 9.5 g CO_2_/(t km) for Russia, intermediate 18 g CO_2_/(t km) for freight trains in Germany, Poland and South Africa and relatively high numbers of 83 g CO_2_/(t km) for freight trains in the US and Colombia^63^.

Ship transport distances were based on imports to Rotterdam, as Germany receives hard coal predominantly via this port. Transport emissions were calculated with a minimum of 4.2 and a maximum of 7.9 g CO_2_/(t nm)^64^.

CO_2_ emissions from mining and beneficiation range from 3.9 to 7.6 g/kWh for the countries considered^65,66^. The highest CO_2_ emissions occurred in the German hard coal mining industry, which is due to the worldwide deepest coal mines. The Polish hard coal industry operates under similar conditions as in Germany and therefore we apply the same range of CO_2_ emissions. The U.S. generally has lower CO_2_ emissions from hard coal mining^67^. In 2018, almost two third of U.S. coal production came from surface mines^68^ and most underground hard coal mines are at rather shallow depths. Low CO_2_ emissions are also estimated for Colombia, as around 90% of the annual hard coal production comes from the large surface mines^69^. For South Africa, we propose slightly higher CO_2_ emissions than for Colombia, as only about 60% comes from opencast mining^70^. Although currently three quarters of the Russian coal production comes from surface mines^71^, we estimate the CO_2_ emissions to be higher than in South Africa because of extreme winter conditions with high energy demand in prominent mining areas. Overall, this results in upstream CO_2_ emissions of ~ 8 g/kWh for domestic and of ~ 12 g/kWh for imported hard coal. Lignite has on average upstream CO_2_ emissions of ~ 10 g/kWh^62^. For comparison, upstream CO_2_ emissions for coal produced and used in the U.S. are estimated at 7 g/kWh^12^.

### Coal in-use CO_2_ emissions

In-use CO_2_ emissions are based on specific net calorific values and carbon contents for the different hard coals^59^ and domestic lignite^72^. Heating values for domestic coals range from 5.8 to 8.4 kWh/kg^59^.

Domestic lignite net calorific heating values are rather low and in the range of 2.2 to 2.9 kWh/kg^58,73^. For calculating specific CO_2_ emissions, we consider the share of lignite with different compositions from the three main lignite mining districts in Germany^56^.

From the carbon content of the individual coals and the net calorific values^59^ and supplemented by additional country-specific data^57,58^, we assess CO_2_ emissions per unit energy for each individual coal. Our calculated mean of ~ 344 g CO_2_/kWh (min: 334.5; max: 354.3) for hard coal used in year 2018 is slightly higher than the Germany-specific value calculated from fuel input and electricity generation of 330 g CO_2_/kWh reported for 2017 by the IEA^74^ and 335 g CO_2_/kWh estimated for 2018 by the UBA^7^. For the specific lignite mix from the three German mining regions in 2018, we calculate ~ 400 g CO_2_/kWh, which is slightly less than the value of 406 g CO_2_/kWh reported by UBA^7^. Corrected for coal plant efficiencies and including supply-chain CO_2_ emissions we calculated 965 g CO_2_/kWh (hard coal 811 g CO_2_/kWh; lignite 1052 g CO_2_/kWh), significantly higher than the mean of 898 g CO_2_/kWh (min 794, max 1003 g CO_2_/kWh) as estimated by Tanaka et al.^18^ for Germany. On the other hand, our calculated emissions are marginally lower than the values reported for 2018 by the UBA^7^ of 835 g CO_2_/kWh for hard coal and 1137 g CO_2_/kWh for lignite, respectively (without supply-chain CO_2_ emissions).

### Natural gas mix Germany

Statistics of natural gas volumes combusted in power plants are not reported on import country specific levels for Germany. The majority of Germany’s natural gas demand is imported, from Russia, Norway and the Netherlands. In 2018 this amounted to 117 Billion (10^9^) m^3^ (bcm) natural gas, plus 7 bcm from domestic production. About one third, or 40 bcm, of this volume was re-exported. Our approach is based on officially reported imports and exports^75^, but presuming that a significant volume of the high-calorific gas from Russia is re-exported. A 10% uncertainty distribution for the country specific natural gas supply proportion for power plants was assigned in the Monte Carlo simulations. However, due to the narrow range (Supplementary Table S1) these variations in gas apportionment have an overall minor effect. The percentages used here (22% NL, 26% NOR, 39% RU, 8% domestic, 6% others) differ only slightly from the values used by Tanaka et al.^18^ of 21% NL, 32% NOR, and 38% RU.

### Methane slip

Another source for methane emissions is the incomplete combustion in gas-fired turbines—so called methane slip. The IPCC recommends to use 4 g CH_4_/GJ (0.014 g CH_4_/kWh) in its global and general guidelines^54^, which would correspond to 0.02% leakage rate in our model for Germany. Newer studies show that methane slip can differ considerably, e.g. aircraft-based measurements of selected natural gas power plants in the U.S. show a large loss range from negative values up to 0.2% of the natural gas used in the power plants^76^. For Germany a single published methane slip value exists (75 g CH_4_/GJ or 0.27 g CH_4_/kWh or 0.4% methane leakage rate)^62^. For power plants in the EU lower values of 1 g CH_4_/GJ (0.004 g CH_4_/kWh), corresponding to 0.005% methane leakage rate from incomplete combustion, have been reported in a more recent study^53^. This large range of 0.005% to 0.4% leakage rates shows the uncertainties in our current understanding of the magnitude of methane slip in Germany. Methane slip from incomplete combustion, although reported as minor driver of emissions additionally contributes to in-use GHG emissions and thus reduces the relative climate benefit for a coal-to-gas switch scenario.

### Natural gas supply chain CO_2_ emissions

Supply chain CO_2_ emissions for domestic and imported natural gas were reported for year 2014^28^ and we assume that these numbers did not change significantly in 2018. Domestic gas emissions are higher than in the Netherlands and Norway, which is mainly due to the energy demands for H_2_S removal from the produced gas in Germany^28^. Natural gas transported from Russia, considering the different pipeline routes to Germany, has supply chain emissions of ~ 46 g CO_2_/kWh, about ten times higher than for gas from the Netherlands with ~ 4 g CO_2_/kWh (Table 2).

### Natural gas in-use CO_2_ emissions

Country specific average compositions of natural gas^58^ and, thus, energy contents were applied to calculate in-use CO_2_ emissions of the gas mix used for power generation in 2018. Calculated in-use CO_2_ emissions for seasonal natural gas from these countries range from 199 to 203 g CO_2_/kWh. Due to the high methane content, in-use CO_2_ emission for natural gas from Russia are lowest.

Using a power plant efficiency of 55% we calculated a mean of ~ 409 g CO_2_/kWh for combined in-use and supply-chain CO_2_ emissions, which is similar to the 399 g CO_2_/kWh estimated by UBA^7^. However, this is significantly larger than estimates by Tanaka et al.^18^ for Germany (mean 342, min 331, max 352 [g CO_2_/kWh]). This difference is primarily explained by the lower gas power plant efficiency applied by us as recently reported^7^, as well as the large share of natural gas from Russia with its relatively high supply-chain CO_2_ emission budget, resulting from the long distance, transcontinental pipeline transport.

## Supplementary Information

Supplementary Information.
