# Supplementary material for: On the climate benefit of a coal-to-gas shift in Germany’s electric power sector
Source: Sci Rep. 2021 Jun 1;11:11453. doi: 10.1038/s41598-021-90839-7 (PMC8169676; doi:10.1038/s41598-021-90839-7)
Supplement: Supplementary file 1 — Supplementary Information. [file 41598_2021_90839_MOESM1_ESM.pdf]

On the climate benefit of a coal-to-gas shift in Germany’s electric power sector

SUPPLEMENT

Stefan Ladage[1], Martin Blumenberg*[1], Dieter Franke*[1], Andreas Bahr[1], Rüdiger Lutz[1], Sandro Schmidt[1]*

[1]Federal Institute for Geosciences and Natural Resources (BGR), Stilleweg 2, 30655 Hannover, Germany;

*Email: Stefan.Ladage@bgr.de; Martin.Blumenberg@bgr.de; Dieter.Franke@bgr.de

22 pages, 5 Figures, 6 Tables

Contents

1 Statistical “Fleet –conversion” modelling 3

2 Compilation of natural gas supply chain methane emission data 10

2.1 Methane emission data for natural gas imported to Germany 10

2.2 Compilation of U.S. nationwide and global methane emission studies 15

2.3 Compilation of regional or basin wide methane emission studies in the U.S. 16

3 Potential LNG import to Germany - supply chain- emissions 17

4 References 19

List of tables

Table S1: Modelling input parameters

Table S2: Monte Carlo simulation GHG emission modelling results

Table S3: Compilation of natural gas leakage rates reported for the Russian Federation, Norway, Netherlands and Germany

Table S4: Published methane leakage rates (global and U.S. estimates)

Table S5: Overview of published methane leakage selected U.S. basins

Table S6: LNG import to Europe: overview of reported GHG emissions.

List of Figures

Figure S1: Input distributions for CO_2 emissions from natural gas

Figure S2: Input distributions for CO_2 emissions from coal

Figure S3: Input distributions for methane emissions from coal

Figure S4: Input distributions for power plant efficiencies by fuel

Figure S5: Comparison of input distributions for CO_2 emissions from natural gas and coal for Germany-specific power plant fuel mixes

1 Statistical "Fleet –conversion" modelling

We modeled the GHG radiative forcing effect of fuel-switching scenario from coal to natural gas in Germany's power sector. This scenario is based on supply chain and in-use CO_2 emissions from the coal and natural gas sector and methane emissions from the coal sector for year 2018. Fuel-specific emissions from the individual supply countries and domestic sources are considered (Table S1). Break-even methane emission rates from natural gas usage are determined by the modeling. Natural, geological and technological variability of the key input parameters were accounted for using Monte Carlo simulations. Truncated distributions were applied for several parameters to achieve physically reasonable simulations.

Few, well constrained parameters (supply chain CO_2 emission for coal and gas, country shares for coal) were modelled with fixed values. Most parameters were presumed to be uncorrelated. The only exception is the efficiency of gas power plants, which has a direct effect on the amount of methane needed for generating a certain amount of energy (e.g., 1 MWh). For these parameters a correlation with r ~ -0.8 was modelled using a Gaussian copula.

The simulations were performed using the software packages Scilab,[1] R[2] and Julia[3]. 100,000 runs were computed for the Monte Carlo simulations. The results are given in Table S2 and selected input parameter distributions are shown (Figures S1- S5)

Table S1: Modelling input parameters. Netherlands (NL), Norway (NOR), Russia (RU), Colombia (CO), Poland (PL), South Africa (ZA), Germany (DE)

Parameter	unit	distribution	mean	standard dev.	truncation min.	truncation max.	remark
CO2 in-use, gas, NL	kg/MWh	truncated normal	200.8	0.5/1.282*(max-min)	200.3	201.2	truncation at P10 and P90
CO2 in-use, gas, NOR	kg/MWh	truncated normal	202.9	0.5/1.282*(max-min)	202.0	203.8	truncation at P10 and P90
CO2 in-use, gas, RU	kg/MWh	truncated normal	198.9	0.5/1.282*(max-min)	198.6	199.1	truncation at P10 and P90
CO2 in-use, gas, others	kg/MWh	truncated normal	200.7	0.5/1.282*(max-min)	200.1	201.2	truncation at P10 and P90
CO2 in-use, gas, DE	kg/MWh	truncated normal	200.1	0.5/1.282*(max-min)	199.5	200.7	truncation at P10 and P90
CO2 supply chain, gas, NL	kg/MWh	fixed value	3.9				
CO2 supply chain, gas, NOR	kg/MWh	fixed value	12.4				
CO2 supply chain, gas, RU	kg/MWh	fixed value	46.1				
CO2 supply chain, gas, others	kg/MWh	fixed value	28.2				
CO2 supply chain, gas, DE	kg/MWh	fixed value	18.5				

share gas, NL	%	uniform	22.1		mean - 10	mean + 10	
share gas, NOR	%	uniform	25.8		mean - 10	mean + 10	
share gas, RU	%	uniform	38.6		mean - 10	mean + 10	
share gas, others	%	uniform	5.5		0	11	
share gas, DE	%	none, fixed value	8				
Mass methane per quantum electricity	kg/MWh	truncated normal	122.3	4.5	0.9 * mean	1.1 * mean	correlated with "Gas … efficiency", r = -0.8
Gas power plant, efficiency	%	truncated normal	55	mean/15	0.9 * mean	1.1 * mean	correlated with "Mass methane …", r = -0.8
hard coal power plant, efficiency	%	truncated normal	44	mean/15	0.9 * mean	1.1 * mean	
lignite power plant, efficiency	%	truncated normal	39	mean/15	0.9 * mean	1.1 * mean	
Coal share hard-coal:	%	fixed value	36.2				
Coal share lignite	%	fixed value	63.8				
share, hard coal, PL	%	fixed value	0.66				
share, hard coal, CO	%	fixed value	10.81				
share, hard coal, RU	%	fixed value	49.57				
share, hard coal, ZA	%	fixed value	2.5				
share, hard coal, USA	%	fixed value	18.02				
share, hard coal, DE	%	fixed value	9.93				
share, hard coal, others	%	fixed value	8.5				
share, lignite, DE-Lusatia	%	fixed value	38.4				
share, lignite, DE-Rhineland	%	fixed value	50.1				
share, lignite, DE-Central Germany	%	fixed value	11.5				
CO2, in-use, hard coal, PL	kg/MWh	truncated normal	349.7	0.5/1.282*(max-min)	342.9	356.4	truncation at P10 and P90
CO2, in-use, hard coal, CO	kg/MWh	truncated normal	358.9	0.5/1.282*(max-min)	339.5	378.3	truncation at P10 and P90
CO2, in-use, hard coal, RU	kg/MWh	truncated normal	350.0	0.5/1.282*(max-min)	343.9	356.0	truncation at P10 and P90
CO2, in-use, hard coal, ZA	kg/MWh	truncated normal	351.4	0.5/1.282*(max-min)	340.7	362.0	truncation at P10 and P90
CO2, in-use, hard coal, USA	kg/MWh	truncated normal	325.4	0.5/1.282*(max-min)	319.7	331.0	truncation at P10 and P90
CO2, in-use, hard coal, DE	kg/MWh	truncated normal	331.0	0.5/1.282*(max-min)	320.1	341.9	truncation at P10 and P90
CO2, in-use, hard coal, others	kg/MWh	truncated normal	349.0	0.5/1.282*(max-min)	319.7	378.3	truncation at P10 and P90
CO2, in-use, lignite, DE-Lusatia	kg/MWh	truncated normal	399.4		min(data) - 0.5*s	max(data) + 0.5*s	
CO2, in-use, lignite, DE-Rhineland	kg/MWh	truncated normal	403.5		min(data) - 0.5*s	max(data) + 0.5*s	
CO2, in-use, lignite, DE-Central Germany	kg/MWh	truncated normal	373.3		min(data) - 0.5*s	max(data) + 0.5*s	
CO2, supply chain, hard coal, PO	kg/MWh	fixed value	9.6				
CO2, supply chain, hard coal, CO	kg/MWh	fixed value	9.98				
CO2, supply chain, hard coal, RU	kg/MWh	fixed value	13.34				
CO2, supply chain, hard coal, ZA	kg/MWh	fixed value	12.65				
CO2, supply chain, hard coal, USA	kg/MWh	fixed value	15.94				
CO2, supply chain, hard coal, DE	kg/MWh	fixed value	7.89				

CO2, supply chain, hard coal, others	kg/MWh	fixed value	11.57				
CO2, supply chain, lignite, DE	kg/MWh	fixed value	10.19				
CH4, hard coal, PL	kg/MWh	truncated normal	1.091	0.5/1.282*(max-min)	0.9871	1.195	truncation at P10 and P90
CH4, hard coal, CO	kg/MWh	truncated normal	0.739	0.5/1.282*(max-min)	0.4971	0.981	truncation at P10 and P90
CH4, hard coal, RU	kg/MWh	truncated normal	0.880	0.5/1.282*(max-min)	0.1861	1.574	truncation at P10 and P90
CH4, hard coal, ZA	kg/MWh	truncated normal	0.277	0.5/1.282*(max-min)	0.2582	0.297	truncation at P10 and P90
CH4, hard coal, USA	kg/MWh	truncated normal	0.333	0.5/1.282*(max-min)	0.1757	0.491	truncation at P10 and P90
CH4, hard coal, DE	kg/MWh	truncated normal	0.636	0.5/1.282*(max-min)	0.3774	0.895	truncation at P10 and P90
CH4, hard coal, others	kg/MWh	truncated normal	0.659	0.5/1.282*(max-min)	0.4136	0.905	truncation at P10 and P90
CH4, lignite, DE	kg/MWh	truncated normal	0.013	0.5/1.282*(max-min)	0.005	0.020	truncation at P10 and P90

Table S2: Monte Carlo simulation GHG emission modelling results for a scenario of German power sector fuel switch from coal to natural gas[a]

Parameter	unit	s	p05	p50	p90
CO2 in-use, gas, NL	kg/MWh	0.3	200.3	200.8	201.2
CO2 in-use, gas, NOR	kg/MWh	0.5	202.2	202.9	203.7
CO2 in-use, gas, RU	kg/MWh	0.1	198.6	198.9	199.1
CO2 in-use, gas, others	kg/MWh	0.3	200.2	200.7	201.1
CO2 in-use, gas, DE	kg/MWh	0.3	199.6	200.1	200.6
share, gas, NL	%	4.9	14.2	22.1	30.0
share, gas, NOR	%	4.9	17.9	25.8	33.8
share, gas, RU	%	5.1	30.7	38.6	47.5
share, gas, others	%	3.1	0.6	5.5	10.3
share, gas, DE	%	0.0	8.0	8.0	8.0
CO2 supply chain+in-use, gas MIX	kg/MWh	0.2	200.2	200.5	200.9
Gas, power plant efficiency	%	2.7	50.5	55.0	59.5
CH4, hard coal, CO	kg/MWh	0.12	0.54	0.74	0.94
CH4, hard coal, DE	kg/MWh	0.13	0.42	0.64	0.85
CH4, hard coal, others	kg/MWh	0.13	0.45	0.66	0.87
CH4, hard coal, PL	kg/MWh	0.05	1.00	1.09	1.18
CH4, hard coal, RU	kg/MWh	0.36	0.30	0.88	1.47
CH4, hard coal, ZA	kg/MWh	0.01	0.26	0.28	0.29
CH4, hard coal, USA	kg/MWh	0.08	0.20	0.33	0.47
CH4, lignite, DE-Lusatia	kg/MWh	0.004	0.006	0.012	0.019
CH4, lignite, DE-Central Germany	kg/MWh	0.004	0.006	0.012	0.019
CH4, lignite, DE-Rhineland	kg/MWh	0.004	0.006	0.013	0.019
CO2 in-use, hard coal, CO	kg/MWh	10.0	342.6	359.0	375.2
CO2 in-use, hard coal, DE	kg/MWh	5.6	321.8	331.0	340.2
CO2 in-use, hard coal, others	kg/MWh	15.2	324.2	349.0	373.8
CO2 in-use, hard coal, PL	kg/MWh	3.5	344.0	349.6	355.3
CO2 in-use, hard coal, RU	kg/MWh	3.1	344.8	349.9	355.1
CO2 in-use, hard coal, ZA	kg/MWh	5.5	342.4	351.4	360.3
CO2 in-use, hard coal, USA	kg/MWh	2.9	320.6	325.3	330.1
CO2 in-use, lignite, DE-Lusatia	kg/MWh	2.7	395.0	399.4	403.8
CO2 in-use, lignite, DE-Central Germany	kg/MWh	4.6	365.4	373.4	380.6
CO2 in-use, lignite, DE-Rhineland	kg/MWh	8.3	389.8	403.5	417.6
CO2 supply chain+in-use, coal MIX	kg/MWh	2.9	385.3	390.0	394.9
CH4 supply-chain, coal MIX	kg/MWh	0.07	0.16	0.27	0.37
CH4 supply-chain eff. coal MIX	kg/MWh	0.15	0.36	0.60	0.85
hard coal, power plant efficiency	%	2.2	40.4	44.0	47.6
lignite, power plant efficiency	%	1.9	35.8	39.0	42.2
CO2 eff. hard coal	kg/MWh	40.9	750.3	812.1	884.7

CO2 eff. lignite	kg/MWh	53.5	967.4	1047.6	1142.3
Normalization of natural gas efficiency	kg/MWh	4.4	115.0	122.3	129.6
CO2 emission, gas MIX	kg/MWh	20.6	377.9	409.3	445.6
CO2 emission, coal MIX	kg/MWh	37.3	905.9	963.3	1027.9
leakage t=1	%	0.39	3.55	4.17	4.85
leakage t=20	%	0.46	4.17	4.89	5.69
leakage t=100	%	0.93	8.37	9.83	11.44

[a]The mean column shows arithmetic mean results and s denotes the standard deviation. pXX: are percentiles (i.e. p05 means that 5 % of the results are smaller than this value). Netherlands (NL), Norway (NOR), Russian Federation (RU), Germany (DE), Colombia (CO), Poland (PL), South Africa (ZA). Time [years] is indicated as t.

Country-specific distributions of CO2 emissions from natural gas and from coal are shown in Figures S1 and S2. The distributions for methane emissions from coal from the different import countries and Germany are shown in Figure S3. The variance in power plant efficiencies is shown in Figure S4. The overall CO_2 emissions of the gas and coal mix, respectively in 2018 in Germany are shown in Figure S5.

It should be noted that about one seventh of the coals declared as steam coal imports were used in the steel industry, mainly as PCI (pulverized coal injection) coals. As no information is available on a corresponding quantity breakdown per country, the percentage ratios by supplier country could not be adapted.

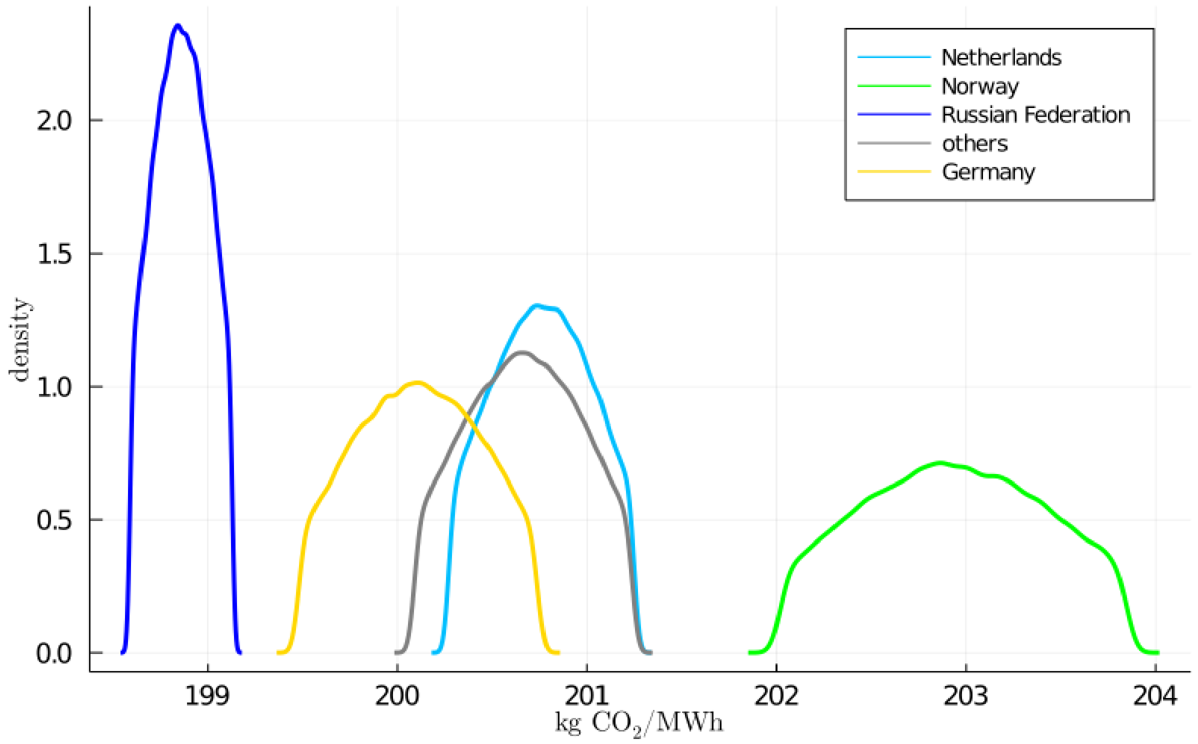

Figure S1: Input distributions for CO_2 emissions from natural gas (by country of origin); NL – Netherlands; NOR – Norway; RU – Russian Federation; DE – Germany, for Monte Carlo simulation.

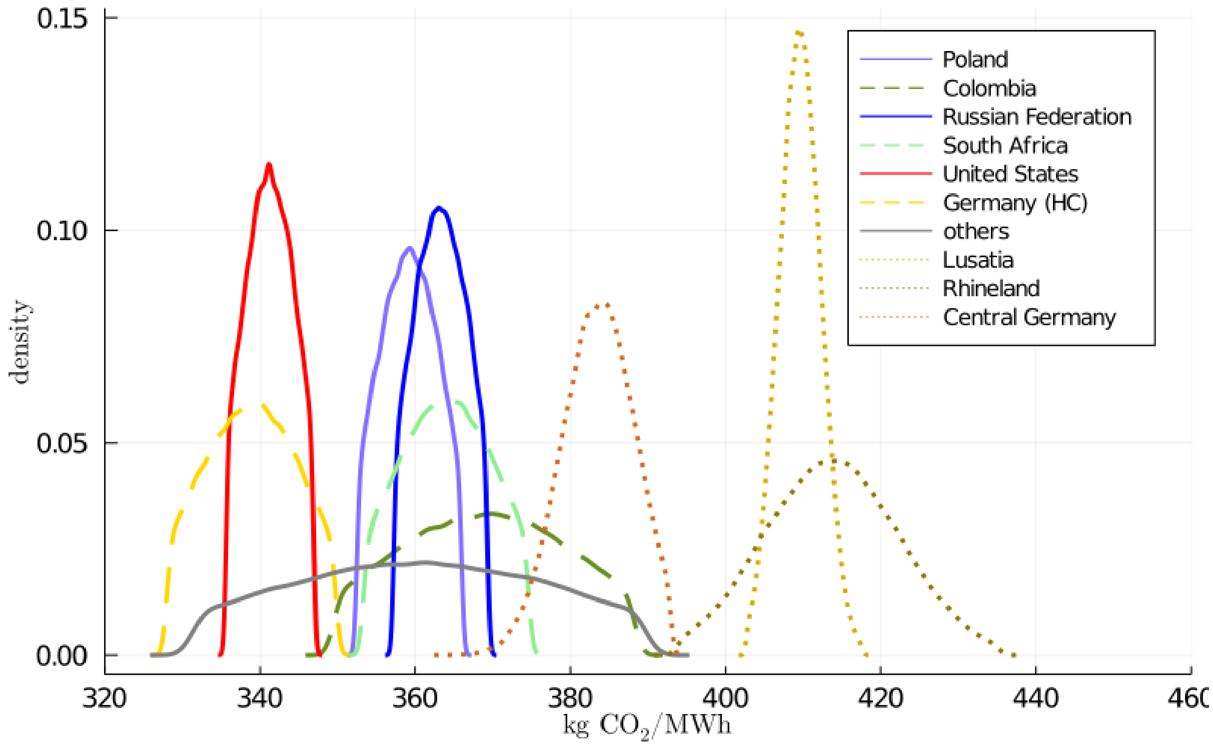

Figure S2: Input distributions for CO_2 emissions from coal (HC - hard coal, imported into Germany and lignite produced in Germany, respectively), for Monte Carlo simulation. Dotted lines indicate lignite.

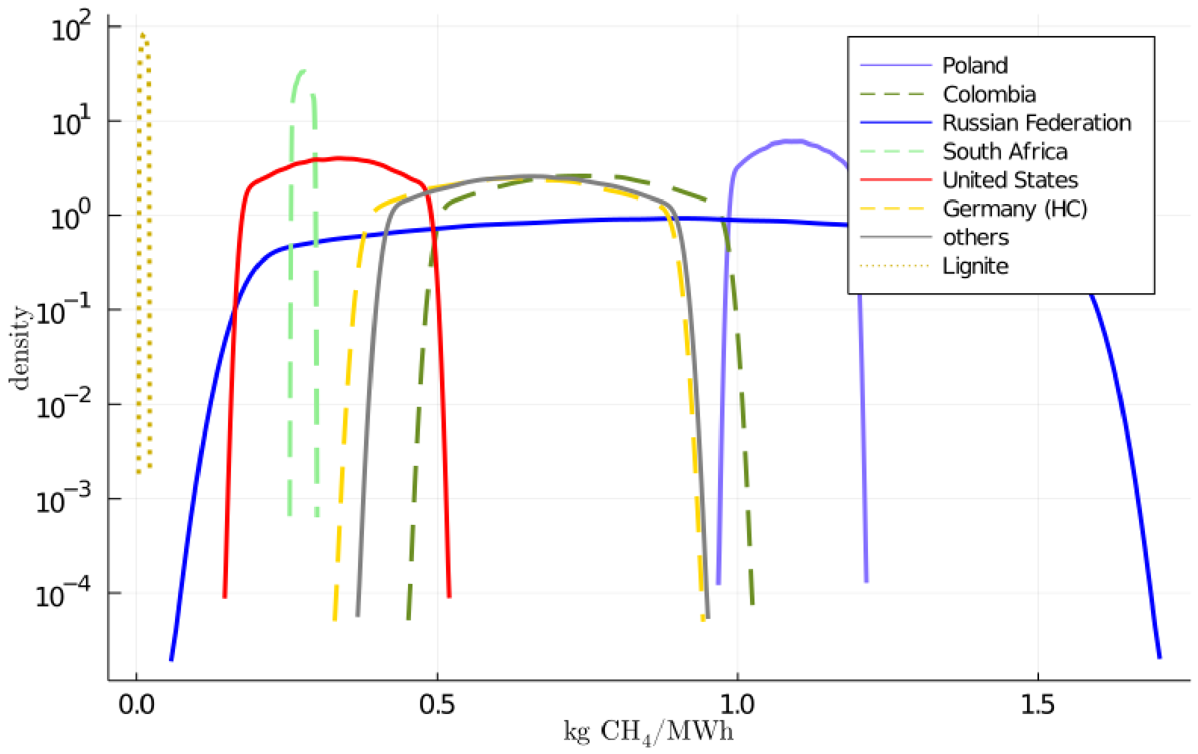

Figure S3: Input distributions for methane emissions from coal (HC - hard coal, imported into Germany and lignite produced in Germany, respectively), for Monte Carlo simulation.

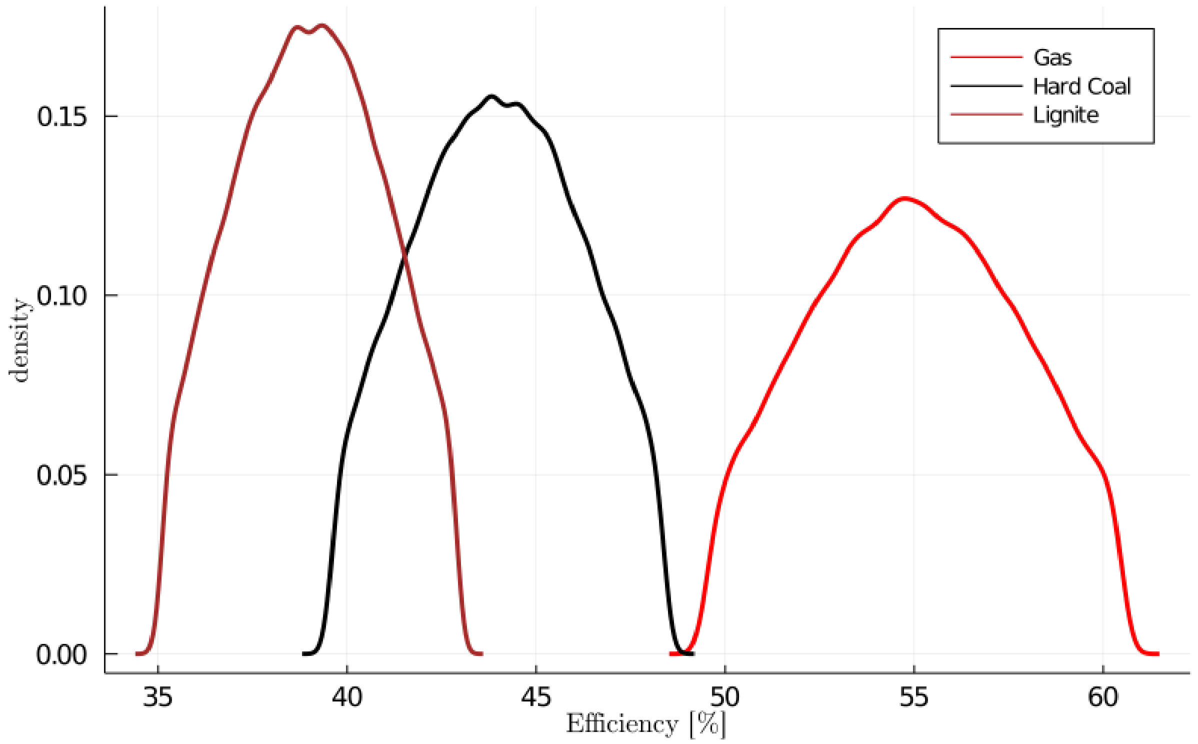

Figure S4: Input distributions for power plant efficiencies by fuel, for Monte Carlo simulation.

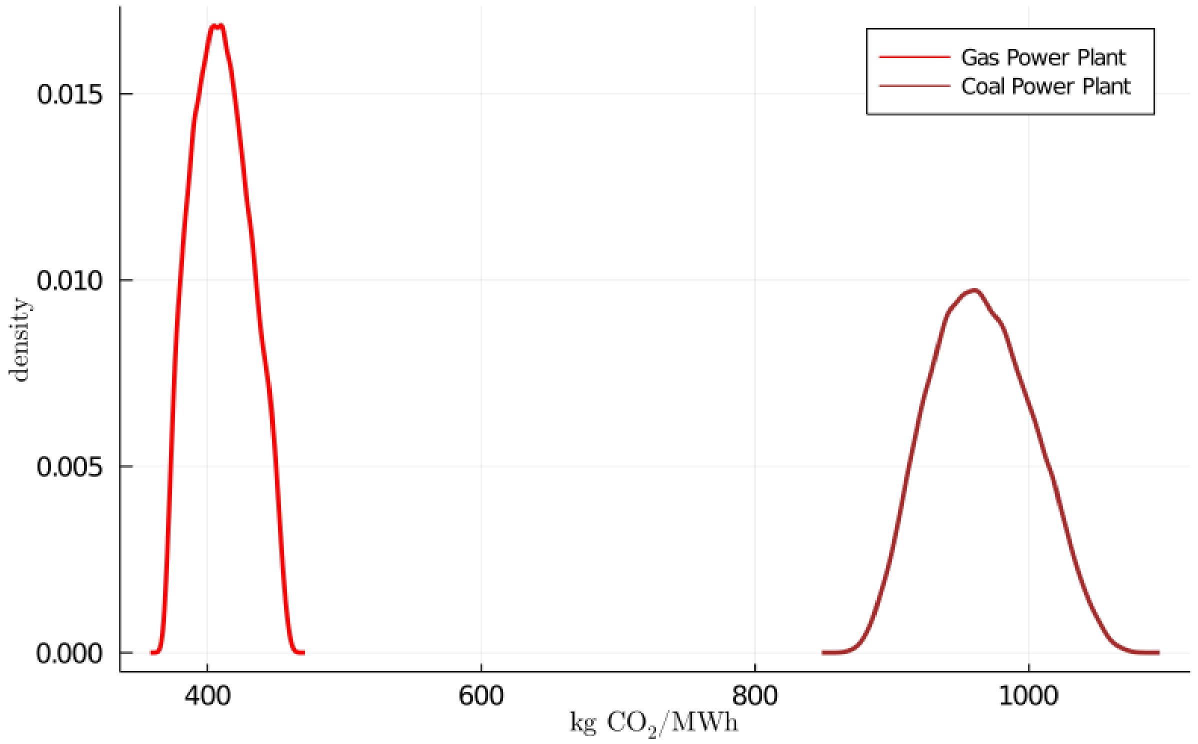

Figure S5: Comparison of input distributions for CO_2 emissions from natural gas and coal (hard coal and lignite) for Germany-specific power plant fuel mixes (including supply-chain and in-use emissions and considering power plant efficiencies, for Monte Carlo simulation.

2 Compilation of natural gas supply chain methane emission data

2.1 Methane emission data for natural gas imported to Germany

Russian Federation

Russian natural gas exported to Europe is primarily produced from fields in West Siberia, as well as from the recently developed fields of the Arctic Yamal Peninsula, where also a modern processing hub has been constructed.

According to the LCA studies[4-6] methane leakage rates for production of natural gas exported to Germany amount to 0.0092 %.[6] This has been reevaluated to 0.016 % based onDBI[5] and – considerably higher to 0.5 % by EXERGIA S.A. et al.[4] (Table S3). The NIR for 2012 reports leakage rates for the production/processing category of 0.54 %,[7] which is in a similar range as reported for the same year by others if applying current efficiencies of German natural gas power plants.[8] Since 2015 the NIR uses a considerably smaller emission factor resulting in a leakage rate of 0.032 %.[9]

The Russian Federation operates a high pressure, long distance trans-continental pipeline infrastructure comprising three export routes, each about 4,000 km long, into Central Europe. Methane leakages along these routes are reported by DBI[5] as a distance weighted mean of 0.26 % of the injected volume. Similar leakage rates are reported in the “thinkstep study”, except 0.17 % for the northern route. This route utilizes the 1,200 km long North Stream pipeline through the Baltic Sea. Under water and without any further compressor facilities this pipeline is considered as a closed system, without any natural gas leakages into the atmosphere.[6]

Already in 2003 – before completion of the North Stream pipeline – a field campaign to study the natural gas leakage rates along the long distance export pipeline routes was conducted by Lechtenböhmer et al.[10]. They assessed leakage rates between 0.5–1.5 %, with a mean of 0.6 %, slightly less than a previous assessment by Dedikov et al.[11], which is based on a more restricted dataset from prior the breakdown of former USSR. Mitigation measures since then led to reductions in natural gas leakages.[12] Overall, according to recent studies, “bottom up” methane leakage rates for Russian pipeline gas to Germany and the EU, respectively, are in the order of 0.3 %. Bearing this in mind and considering that further leakage reduction mitigation measures have since been implemented as well as that the first North Stream pipeline has gone in operation, it appears that gas leakage rates of 1 %[4] for Russian pipeline transport are probably rather high.

Table S3: Compilation of natural gas leakage rates reported for the Russian Federation, Norway, Netherlands and Germany

Report/ Study	Data reference year	CH_4 leakage rate [%]			
		Production	Processing	Long distance transport	Sum supply chain
**Russian Federation**					
EXERGIA S.A. et al.[4]	2012	0.5000	n.s.[a]	1	1.500
DBI[5]	2014	0.0160	n.s.[a]	0.26	0.276
thinkstep AG[6 b]	2015	0.0092	n.s.[a]	0.3[c]	0.309
Höglund-Isaakson [8]	2012	0.65	-	-	-
Lechtenböhmer et al.[12d]	2003	n.s.	n.s.	0.6	n.s.
NIR 2020 [9]	2018	0.032	0 [a]	(1.0) [e+f]	(1.032)
**Norway**					
EXERGIA S.A. et al.[4]	2012	0.00500	0.005	0.00028	0.010
DBI[5]	2014	0.00800	0.0080	0	0.016
thinkstep AG[6]	2015	0.00458	0.0042	0	0.009
NIR 2020 [13]	2018	0	0	0 [e]	0.003
**Netherlands**					
EXERGIA S.A. et al.[4]	2012	0.03	0	0.00028	0.030
DBI[5]	2014	0.026	0	0.000114	0.026
thinkstep AG[6]	2014	0.026	0.0017	0.000114	0.028
NIR 2020 [14]	2018	0	0	0.019[9]	0.019
**Germany (domestic)**					
DBI[5]	2014	0.0189	0.016	0.001618[g]	0.036518[h]
NIR 2020 [15]	2018	0.008 [i]	n.s.[a]	n/a	0.246 [k]
**GasMix to Germany [l]**					
UBA 2017 [16]	2016				0.71 [m]

[a]: processing not reported separately, either included in production or transport net; [b]: primarily based on DBI values; [c]: unweighted, global mean deduced from reported individual transport route values; [d]: duplications from further publications based on the same dataset i.e Lechtenböhmer et al.[10,] Lelieveld et al.[17] are not listed here; [e]: leakage rate resembles rubric 1.B.2.B.iv (transmission) of the NIR, [f]: comprises emission of the intra Russian distribution network, not representative for high-pressure long distance pipelines. [g]: intra German distribution and transport; [h]: sum intra German supply chain; [i]: leakage rate domestic production Germany; [k]: leakage rate normalized to sum of consumed and transmitted in Germany. [l]: GasMix from different import countries to Germany. [m]: calculated from 0.48 g CH_4/kWh considering 67.3 g CH_4 to generate 1 kWh in a natural gas plant in Germany. Reports in this table other than “NIR” are taken as basis for calculating leak rates for the “Other Reports” scenario in Figure. 4; n.s.: not specified.

Norway

Norway's natural gas resources are located offshore on the Norwegian continental shelf. Natural gas is primarily found and produced as associated gas from oil fields. Notable exceptions are the Troll Field (North Sea) and Snøhvit (Barents Sea), which predominantly produce dry natural gas. In general, the natural gas is processed offshore and injected into the North Sea offshore pipeline network, directly transporting natural gas to the EU customers. In case of Snøhvit, however, natural gas is exported via LNG.

Methane leakage rates for offshore production and processing, respectively have been reported before 2014 unanimously in various studies around or less than 0.006 % (Table S3). Updated data since 2014 led to an order of magnitude reduced rates of approx. 0.0004 %.[5, 6] Similarily, the Norwegian Environment Agency (NEA) observed that methane emissions are considerably lower than previously assessed.[18]

Underwater pipelines are considered a closed system, which generally inhibits atmospheric methane emissions.[5, 6] The NEA, however, uses the emission factor of 0.0003 % for the pipeline transport network, following the IPCC 2006 protocol,[19] corresponding to another report.[4]

Overall, methane leakage rates for natural gas from offshore Norway to Germany are reported to be less than 0.02 %.

Netherlands

Natural gas from the Netherlands supplied to Germany comes primarily from the Groningen gas field, west of the German-Dutch border. For production and processing, leakage rates of approx. 0.03 % are reported (Table S3). Due to the short distance, leakage rates for transport are less than 0.0001 %.[4-6] Updates of the methane emission factors have led to lower transport leakage rates in the more recent studies. Overall, methane leakage rates for natural gas from the Netherlands to Germany are reported to be less than 0.03 %.

Yacovitch et al.[20] performed measurements on methane emissions from the natural gas supply chain for a region in the Netherlands, including the Groningen natural gas field. Although the methane emissions determined were low when compared to production sector emissions in the U.S., the authors report that CH_4 emissions exceed the Netherlands NIR data. Uncertainties in their measurements are, however, large and the resulting error range includes the current NIR value of 0.03 %.[14]

Nevertheless, even assuming a factor 10 higher emission rate for the Groningen field would still yield an overall low emission rate compared to other suppliers for natural gas to Germany and EU, respectively.

Germany

Domestic production of natural gas is mainly concentrated in the North of Germany (Federal State of Lower Saxony). According to the published DBI report methane leakage rates for domestic gas are low (0.04 % for 2014[5]). The most recent National Inventory Report reported 10 fold higher values, but this also includes transmitted gas through Germany (Table S3).[15] A recent exemplary study on methane losses in the local natural gas distribution network of Hamburg, Germany found loss rates of 0.04 – 0.07 % of total annual natural gas consumption of Hamburg.[21]

2.2 Compilation of U.S. nationwide and global methane emission studies

Table S4: Published methane leakage rates (global and U.S. estimates) [a]

Source	Scope/ region/ comment	Reference year	CH_4 emissions in the natural gas supply chain		
			Shale gas	Conventional gas	Total
**Global**					
Hayhoe et al. (2002)[22]	Global summary. (according to GRI (1997))	probably 1997			0.5 % production + 2.5 % distribution and usage (0.76 kg/GJ)
Schwietzke et al. (2016)[23]	Global estimate based on atmospheric $\delta^{13}C$-methane	2013			2.2 %
World Energy Outlook (2017)[24]	Global estimate (data source unpublished)	2015			1.7 %
**USA wide**					
Howarth et al. (2011)[25]	Shale gas, conventional natural gas USA (questioned by e.g.[26])	2007	3.6–7.9 %	1.7–6 %	
Hultman et al. (2011)[27]	Shale gas, tight gas, CBM USA	2007	2.8 % production + 0.9 % downstream	1.3 % production + 0.9 % downstream	
Burnham et al. (2012)[28]	USA	2009	2.01% (0.71–5.23 %)	2.75 % (0.92–5.47 %)	
Cathles et al. (2012)[26]	USA; numbers are based on EPA 2009 and calculations (no measurements)	2008	1.1 % (0.2 % production + 0.9 % distribution and processing)		0.9–2.2 %
Littlefield et al. (2017)[29]	USA	2012			1.7 % (1.3–2.2 % at 95 % confidence)
EPA (2017)[30]	USA	2015			1.4 % (*)
Alvarez et al. (2018)[31]	Based on measure-ments in ~30% of natural gas production regions in the USA	2015			2.3 % (1.9–2.6%)
Omara et al. (2018)[32]	USA natural gas production. (Downstream from[31])	2016			1.5 % (non-parametric model) and 0.59 % (regression model) natural gas production without 1 % downstream emissions
EPA (2020)[33]	USA (calculated using production numbers from [34])	2018			1.1 (1.3) %

[a]calculated based on a production of 523 x 10^6 t(CH_4)/yr (90 % methane out of 581 x 10^6 t natural gas production) in 2018. https://www.eia.gov/dnav/ng/ng_prod_sum_a_EPG0_FPD_mmcf_a.htm; bracketed values include CH_4 emissions from oil production.

2.3 Compilation of regional or basin wide methane emission studies in the U.S.

Table S5: Overview of published methane leakage studies of selected U.S. basins

Reference	Region/comment	Year	CH_4 loss rates		
USA (basin /well pads)			Shale gas	Conventional gas	Total
Peischl et al. (2015)[35]	Marcellus Shale (Pennsylvania + W Virginia)	2013	0.18–0.41 %		
Omara et al. (2016)[36]	Marcellus Shale (Pennsylvania + W Virginia)	2014/ 2015	0.13 % (0.01–1.2%)	10.5 % (0.35–91%)	
Omara et al. (2018)[32]	Marcellus Shale (Pennsylvania + W Virginia)	2016			Production: 0.27 %
Ren et al. (2019)[37]	Marcellus Shale (Pennsylvania + W Virginia)	2015	1.1 % (0–3.5%)		
Peischl et al. (2015)[35]	Haynesville (Arkansas, Louisiana, East Texas)	2013	1.0–2.1%		
Peischl et al. (2018)[38]	Haynesville (Arkansas, Louisiana, East Texas)	2015	1.0 %		
Karion et al. (2015)[39]	Barnett Shale (Texas)	2013	1.3–1.9 %		
Lyon et al. (2015)[40]	Barnett Shale (Texas)	2013	1.1 % (1.0–1.3 %) (4% oil production not considered)		
Zavala-Araiza et al. (2015)[41]	Barnett Shale (Texas)	2013	1.2–1.9 %		
Peischl et al. (2018)[38]	Barnett Shale (Texas)	2015	1.5 %		
Omara et al. (2018)[32]	Barnett Shale (Texas)	2016			Production: 0.65 %
Schneising et al. (2014)[42]	Bakken (North Dakota etc.)	2006-2008 (a)<br>2009-2011 (b)	a) 10.1 % (± 7.3 %)<br>9.1 % (± 6.2 %)		
Peischl et al. (2016)[43]	Bakken (North Dakota etc.)	2014	4.2–8.4 %		
Peischl et al. (2018)[38]	Bakken (North Dakota etc.)	2015	5.4 %		
Peischl et al. (2018)[38]	Eagle Ford East	2015	3.2 %		
Peischl et al. (2018)[38]	Eagle Ford West	2015	2.0 %		
Peischl et al. (2015)[35]	Fayetteville Region	2013	1.0–2.8 %		
Robertson et al. (2017)[44]	Fayetteville (Arkansas)	2015			Production: 0.09 % (0.05−0.15 %)
Omara et al. (2018)[32]	Fayetteville (Arkansas)	2016			Production: 0.5 %
Pétron et al. (2014)[45]	Denver-Julesburg (Colorado)	2012	4% (± 1.5 %)		
Omara et al. (2018)[32]	Denver-Julesburg (Colorado)	2016			Production: 1.6 %
Robertson et al. (2017)[44]	Denver-Julesburg (Colorado)	2014			Production: 2.1 % (1.1−3.9%)
Peischl et al. (2018)[38]	Denver Basin	2015	2.1 %		
Karion et al. (2013)[46]	Uintah County, (Utah)	2012			8.9 % (6.2–11.7 %)
Robertson et al. (2017)[44]	Uintah County, (Utah)	2015			Production: 2.8 % (1.0−8.6%)
Omara et al. (2018)[32]	Uintah County, (Utah)	2016			Production: 3.5 %

Robertson et al. (2017)[44]	Upper Green River (Wyoming)	2014		Production: 0.18 % (0.12−0.29%)
Omara et al. (2018)[32]	Upper Green River (Wyoming)	2016		Production: 0.5 %
Omara et al. (2018)[32]	Pinedale (Wyoming)	2016		Production: 0.65 %
Peischl et al. (2015)[35]	W. Arkoma (Arkansas)	2013	6–20 %	
Zhang et al. (2020)[47]	Permian Basin (Texas & SE New Mexico)	2018/ 2019		Production: 3.7 %

3 Potential LNG import to Germany - supply chain- emissions

Liquefaction, ocean transport and regasification are all energy-consuming processes, further adding CO_2 and CH_4 emissions to the natural gas supply chain for a potential future LNG supply of natural gas from the U.S. or other countries to Germany. Energy demand depends among other factors on the liquefaction process, scaling of the plant, composition of natural gas, ambient temperatures and tanker size. Shipment distances and vessel efficiency are main factors for the energy demand. In total, energy demand for LNG, including liquefaction, transport and regasification, lies between 6 % and 20 % of the gross natural gas amount transported.[48] When considering the entire life cycle of natural gas used for electricity generation, emissions from LNG specific operations are less than 11 % of the overall GHG emissions.[49, 50] Using electricity from renewables and waste heat recovery can reduce these emissions considerably.[48-51]

Additionally, methane slip adds to GHG emissions during transport with ocean going LNG carriers equipped with gas-fired engines. Mallapragada et al.[50] suggested a value of 2 g CH_4 emitted/kg (0.2 %) LNG fuel and calculated a contribution of shipping of 2 % to total GHG emissions of generated electricity. Abrahams et al.[49] calculated the same range (1-3 %).

Overall methane loss rates by LNG specific steps add about 0.2 – 0.4 % to the natural gas supply chain emissions (Table S6).[52] However, a part of this leakage is counterbalanced by excluding emissions from the downstream national distribution grids, which are not utilized when transporting gas to the LNG export terminals. For the U.S. this is in the order of magnitude of ~0.1 % local distribution and ~0.3 % transmission and storage of annually produced gas in 2018.[34]

Table S6: LNG import to Europe: overview of reported GHG emissions*

Study	GHG emissions (g CO_2eq/kWh)	CH_4 leakage rate (%)
Thinkstep (2017), AR4 (25) [a] [6]	13.4–53.6	0.23–<<0.86[f]
Thinkstep (2019), AR5 (30) [53]	44.6	
Mallapragada et al. (2018), AR5 (30) [b] [50]	36	0.04–0.58
Balcombe et al. (2016), AR5 (34)[c] [52]	14-73.1 (median 32.1)	0.2–0.4
Abrahams et al (2015), AR5 (36) [49]	33[e]	
Umweltbundesamt (2019)[d], AR4 (25) [54]	31.7	
NETL (2019)[55], AR5 (36)	69.8	

*Study name and GWPs are given in the first column. GHG emissions for the LNG supply chain, i.e. liquefaction, ocean transport and regasification. Methane leakage rates of produced gas attributed to the LNG supply chain. [a]: „EU-28 Natural Gas Consumption Mix 2015“ (without USA) import to EU North; wide range of values includes natural gas production; [b]: Marcellus Shale to EU (UK, Spain); [c]: comprehensive compilation of estimated CO2 and methane emissions with a focus on the U.S.; [d]: LNG from the U.S. [e]: Median values from Figure S2 in Abrahams et al. (2015); [f]: Includes natural gas production, processing, ocean transport and liquefaction.

Based on the available literature (Table S6) the range of possible methane loss rates of the U.S. supply chain including the LNG supply-chain to Europe appears to be between 1.3 % and 2.5 %. The lower limit corresponds to estimates of EPA[56] and the upper limited to estimations by Alvarez et al.[31], excluding national distribution, but adding the approximate median value for losses reported from the LNG supply chain.[52] Thus, transportation related methane loss rates of potential U.S. LNG transported to Germany on one side and for long distance pipeline gas transport e.g. from Russia on the other side, may both add up to less than 0.4 % leakage rate (Tables S3 and S4). Considering also carbon dioxide emissions for the energy intense LNG or long-distance pipeline transport, does not change the magnitudes of emissions. While the LNG supply chain emissions (liquefaction, ocean transport, regasification) from the U.S. are on average about 36 g CO_2eq/kWh (13.4-73.1 g CO_2eq/kWh, Table S6), pipeline transport from Russia to Germany are about 32-47 g CO_2eq/kWh.[5, 57]

4 References

1. Scilab Enterprises *Scilab free and open source software for numerical computation*, Scilab Enterprises: Orsay, France, 2014.
2. R Core Team, *R: A Language and Environment for Statistical Computing*. R Foundation for Statistical Computing: Vienna, Austria, 2013,
3. Bezanson, J.; Edelman, A.; Karpinski, S.; Shah, V. B., Julia: A Fresh Approach to Numerical Computing. *SIAM Review* **2017,** *59*, (1), 65–98, doi:10.1137/141000671.
4. EXERGIA S.A.; E3M-Lab; COWI A/S *Study on actual GHG data for diesel, petrol, kerosene, and natural gas - Final Report*; ENER/C2/2013-643; European Comission DG ENER: Brussels, 2015, https://ec.europa.eu/energy/studies/study-actual-ghg-data-diesel-petrol-kerosene-and-natural-gas_en?redir=1
5. DBI *Kritische Überprüfung der Default-Werte der Treibhausgasvorkettenemissionen von Erdgas. Abschlussbericht*; 2016, https://www.dbi-gut.de/emissionen.html?file=files/HIPS_net/Emissionen/Finale%20Dokumente/Management_Summary_THG-Vorkettenemissionen_von_Erdgas.pdf
6. thinkstep AG *Greenhouse Gas Intensity of Natural Gas - Final Report*; 2017; p 176, https://www.thinkstep.com/content/report-greenhouse-gas-intensity-study-natural-gas
7. UNFCCC, Russian Federation - National inventory submission 2014 - Common Reporting Format (CRF) Table. In UNFCCC: 2014.
8. Höglund-Isaksson, L., Bottom-up simulations of methane and ethane emissions from global oil and gas systems 1980 to 2012. *Environmental Research Letters* **2017,** *12*, (2), 024007, doi:10.1088/1748-9326/aa583e.
9. UNFCCC, Russian Federation - National inventory submission 2020 - Common Reporting Format (CRF) Table. In UNFCCC: 2020.
10. Lechtenböhmer, S.; Dienst, C.; Fischedick, M.; Hanke, T.; Langrock, T.; Assonov, S. S.; Brenninkmeijer, C. A. M. *Treibhausgasemissionen des russischen Erdgas-Exportpipeline-Systems*; Wuppertal Institut für Klima, Umwelt, Energie GmbH: 2005,
11. Dedikov, J. V.; Akopova, G. S.; Gladkaja, N. G.; Piotrovskij, A. S.; Markellov, V. A.; Salichov, S. S.; Kaesler, H.; Ramm, A.; Müller von Blumencron, A.; Lelieveld, J., Estimating methane releases from natural gas production and transmission in Russia. *Atmospheric Environment* **1999,** *33*, (20), 3291-3299, doi:10.1016/S1352-2310(98)00388-4.
12. Lechtenböhmer, S.; Dienst, C.; Fischedick, M.; Hanke, T.; Fernandez, R.; Robinson, D.; Kantamaneni, R.; Gillis, B., Tapping the leakages: Methane losses, mitigation options and policy issues for Russian long distance gas transmission pipelines. *International Journal of Greenhouse Gas Control* **2007,** *1*, (4), 387-395, doi:https://doi.org/10.1016/S1750-5836(07)00089-8.
13. UNFCCC, Norway - National inventory submission 2020 - Common Reporting Format (CRF) Table. In UNFCCC: 2020.
14. UNFCCC, Netherlands - National inventory submission 2020 - Common Reporting Format (CRF) Tables. In UNFCCC: 2020.
15. UNFCCC, Germany - National inventory submission 2020 - Common Reporting Format (CRF) Table In UNFCCC: 2020.
16. Icha, P.; Kuhs, G. *Entwicklung der spezifischen Kohlendioxid-Emissionen des deutschen Strommix in den Jahren 1990-2016*; UBA - Umweltbundesamt: Dessau-Roßlau, Germany, 2017; p 28,
17. Lelieveld, J.; Lechtenböhmer, S.; Assonov, S. S.; Brenninkmeijer, C. A. M.; Dienst, C.; Fischedick, M.; Hanke, T., Low methane leakage from gas pipelines. *Nature* **2005,** *434*, (7035), 841-842, doi:10.1038/434841a.
18. Husdal, G.; Osenbroch, L.; Yetkinoglu, Ö.; Østebrøt, A. *Cold venting and fugitive emissions from Norwegian offshore oil and gas activities–summary report*; M-515|2016; Add Novatech AS: 12.4.2016, 2016; p 26, https://www.miljodirektoratet.no/publikasjoner/2016/juni-2016/cold-venting-and-fugitive-emissions-from-norwegian-offshore-oil-and-gas-activities--summary-report/
19. IPCC, IPCC Guidelines for National Greenhouse Gas Inventories, Vol. 2, Chapter 4: Fugitive Emissions. In 2006; Vol. 2.
20. Yacovitch, T. I.; Neininger, B.; Herndon, S. C.; van der Gon, H. D.; Jonkers, S.; Hulskotte, J.; Roscioli, J. R.; Zavala-Araiza, D., Methane emissions in the Netherlands: The Groningen field. *Elem Sci Anth* **2018,** *6*, 57, doi:DOI: https://doi.org/10.1525/elementa.308.
21. Maazallahi, H.; Fernandez, J. M.; Menoud, M.; Zavala-Araiza, D.; Weller, Z. D.; Schwietzke, S.; von Fischer, J. C.; Denier van der Gon, H.; Röckmann, T., Methane mapping, emission quantification and attribution in two European cities; Utrecht, NL and Hamburg, DE. *Atmos. Chem. Phys. Discuss.* **2020,** *2020*, 1-30, doi:10.5194/acp-2020-657.

22. Hayhoe, K.; Kheshgi, H. S.; Jain, A. K.; Wuebbles, D. J., Substitution of Natural Gas for Coal: Climatic Effects of Utility Sector Emissions. *Climatic Change* **2002,** *54*, (1), 107-139, doi:10.1023/a:1015737505552.
23. Schwietzke, S.; Sherwood, O. A.; Bruhwiler, L. M.; Miller, J. B.; Etiope, G.; Dlugokencky, E. J.; Michel, S. E.; Arling, V. A.; Vaughn, B. H.; White, J. W.; Tans, P. P., Upward revision of global fossil fuel methane emissions based on isotope database. *Nature* **2016,** *538*, (7623), 88-91, doi:10.1038/nature19797.
24. IEA *World Energy Outlook 2017*; International Energy Agency: London, 2017,
25. Howarth, R. W.; Santoro, R.; Ingraffea, A., Methane and the greenhouse-gas footprint of natural gas from shale formations. *Climatic Change* **2011,** *106*, (4), 679, doi:10.1007/s10584-011-0061-5.
26. Cathles, L. M.; Brown, L.; Taam, M.; Hunter, A., A commentary on “The greenhouse-gas footprint of natural gas in shale formations” by R.W. Howarth, R. Santoro, and Anthony Ingraffea. *Climatic Change* **2012,** *113*, (2), 525-535, doi:10.1007/s10584-011-0333-0.
27. Hultman, N.; Rebois, D.; Scholten, M.; Ramig, C., The greenhouse impact of unconventional gas for electricity generation. *Environmental Research Letters* **2011,** *6*, (4), 049504, doi:10.1088/1748-9326/6/4/049504.
28. Burnham, A.; Han, J.; Clark, C. E.; Wang, M.; Dunn, J. B.; Palou-Rivera, I., Life-cycle greenhouse gas emissions of shale gas, natural gas, coal, and petroleum. *Environmental science & technology* **2012,** *46*, (2), 619-27, doi:10.1021/es201942m.
29. Littlefield, J. A.; Marriott, J.; Schivley, G. A.; Skone, T. J., Synthesis of recent ground-level methane emission measurements from the U.S. natural gas supply chain. *Journal of Cleaner Production* **2017,** *148*, 118-126, doi:10.1016/j.jclepro.2017.01.101.
30. EPA - United States Environmental Protection Agency *Inventory of U.S. Greenhouse Gas Emissions and Sinks - 1990-2015*; EPA 430-P-17-001; EPA: 2017,
31. Alvarez, R. A.; Zavala-Araiza, D.; Lyon, D. R.; Allen, D. T.; Barkley, Z. R.; Brandt, A. R.; Davis, K. J.; Herndon, S. C.; Jacob, D. J.; Karion, A.; Kort, E. A.; Lamb, B. K.; Lauvaux, T.; Maasakkers, J. D.; Marchese, A. J.; Omara, M.; Pacala, S. W.; Peischl, J.; Robinson, A. L.; Shepson, P. B.; Sweeney, C.; Townsend-Small, A.; Wofsy, S. C.; Hamburg, S. P., Assessment of methane emissions from the U.S. oil and gas supply chain. *Science* **2018,** *361*, 186-188, doi:10.1126/science.aar7204.
32. Omara, M.; Zimmerman, N.; Sullivan, M. R.; Li, X.; Ellis, A.; Cesa, R.; Subramanian, R.; Presto, A. A.; Robinson, A. L., Methane emissions from natural gas production sites in the United States: Data synthesis and national estimate. *Environmental science & technology* **2018**, doi:10.1021/acs.est.8b03535.
33. EPA *Inventory of U.S. Greenhouse Gas Emissions and Sinks - 1990-2018*; EPA 430-R-20-002; EPA - United States Environmental Protection Agency,: Washington DC, 13.4.2020, 2020; p 733, https://www.epa.gov/ghgemissions/inventory-us-greenhouse-gas-emissions-and-sinks-1990-2018
34. UNFCCC, United States of America - National inventory submission 2020 - Common Reporting Format (CRF) Table. In UNFCCC: 2020.
35. Peischl, J.; Ryerson, T. B.; Aikin, K. C.; de Gouw, J. A.; Gilman, J. B.; Holloway, J. S.; Lerner, B. M.; Nadkarni, R.; Neuman, J. A.; Nowak, J. B.; Trainer, M.; Warneke, C.; Parrish, D. D., Quantifying atmospheric methane emissions from the Haynesville, Fayetteville, and northeastern Marcellus shale gas production regions. *Journal of Geophysical Research: Atmospheres* **2015,** *120*, (5), 2119-2139, doi:doi:10.1002/2014JD022697.
36. Omara, M.; Sullivan, M. R.; Li, X.; Subramanian, R.; Robinson, A. L.; Presto, A. A., Methane Emissions from Conventional and Unconventional Natural Gas Production Sites in the Marcellus Shale Basin. *Environmental science & technology* **2016,** *50*, (4), 2099-107, doi:10.1021/acs.est.5b05503.
37. Ren, X.; Hall, D. L.; Vinciguerra, T.; Benish, S. E.; Stratton, P. R.; Ahn, D.; Hansford, J. R.; Cohen, M. D.; Sahu, S.; He, H.; Grimes, C.; Fuentes, J. D.; Shepson, P. B.; Salawitch, R. J.; Ehrman, S. H.; Dickerson, R. R., Methane Emissions from the Marcellus Shale in Southwestern Pennsylvania and Northern West Virginia Based on Airborne Measurements. *Journal of Geophysical Research: Atmospheres* **2019,** *124*, (3), 1862-1878, doi:10.1029/2018JD029690.
38. Peischl, J.; Eilerman, S. J.; Neuman, J. A.; Aikin, K. C.; de Gouw, J. A.; Gilman, J. B.; Herndon, S. C.; Nadkarni, R.; Trainer, M.; Warneke, C.; Ryerson, T. B., Quantifying Methane and Ethane Emissions to the Atmosphere From Central and Western U.S. Oil and Natural Gas Production Regions. *Journal of Geophysical Research: Atmospheres* **2018,** *123*, (14), 7725-7740, doi:10.1029/2018JD028622.
39. Karion, A.; Sweeney, C.; Kort, E. A.; Shepson, P. B.; Brewer, A.; Cambaliza, M.; Conley, S. A.; Davis, K.; Deng, A.; Hardesty, M.; Herndon, S. C.; Lauvaux, T.; Lavoie, T.; Lyon, D.; Newberger, T.; Pétron, G.; Rella, C.; Smith, M.; Wolter, S.; Yacovitch, T. I.; Tans, P., Aircraft-Based Estimate of Total Methane Emissions from the Barnett Shale Region. *Environmental science & technology* **2015,** *49*, (13), 8124-8131, doi:10.1021/acs.est.5b00217.

40. Lyon, D. R.; Zavala-Araiza, D.; Alvarez, R. A.; Harriss, R.; Palacios, V.; Lan, X.; Talbot, R.; Lavoie, T.; Shepson, P.; Yacovitch, T. I.; Herndon, S. C.; Marchese, A. J.; Zimmerle, D. J.; Robinson, A. L.; Hamburg, S. P., Constructing a Spatially Resolved Methane Emission Inventory for the Barnett Shale Region. *Environmental science & technology* **2015,** *49*, (13), 8147-8157, doi:10.1021/es506359c.
41. Zavala-Araiza, D.; Lyon, D. R.; Alvarez, R. A.; Davis, K. J.; Harriss, R.; Herndon, S. C.; Karion, A.; Kort, E. A.; Lamb, B. K.; Lan, X.; Marchese, A. J.; Pacala, S. W.; Robinson, A. L.; Shepson, P. B.; Sweeney, C.; Talbot, R.; Townsend-Small, A.; Yacovitch, T. I.; Zimmerle, D. J.; Hamburg, S. P., Reconciling divergent estimates of oil and gas methane emissions. *Proceedings of the National Academy of Sciences* **2015,** *112*, (51), 15597-15602, doi:10.1073/pnas.1522126112.
42. Schneising, O.; Burrows, J. P.; Dickerson, R. R.; Buchwitz, M.; Reuter, M.; Bovensmann, H., Remote sensing of fugitive methane emissions from oil and gas production in North American tight geologic formations. *Earth's Future* **2014,** *2*, (10), 548-558, doi:10.1002/2014ef000265.
43. Peischl, J.; Karion, A.; Sweeney, C.; Kort, E. A.; Smith, M. L.; Brandt, A. R.; Yeskoo, T. W.; Aikin, K. C.; Conley, S. A.; Gvakharia, A.; Trainer, M.; Wolter, S.; Ryerson, T. B., Quantifying atmospheric methane emissions from oil and natural gas production in the Bakken shale region of North Dakota. *Journal of Geophysical Research: Atmospheres* **2016,** *121*, (10), 6101-6111, doi:doi:10.1002/2015JD024631.
44. Robertson, A. M.; Edie, R.; Snare, D.; Soltis, J.; Field, R. A.; Burkhart, M. D.; Bell, C. S.; Zimmerle, D. J.; Murphy, S. M., Variation in Methane Emission Rates from Well Pads in Four Oil and Gas Basins with Contrasting Production Volumes and Compositions. *Environmental science & technology* **2017,** *51*, (15), 8832-8840, doi:10.1021/acs.est.7b00571.
45. Pétron, G.; Karion, A.; Sweeney, C.; Miller, B. R.; Montzka, S. A.; Frost, G. J.; Trainer, M.; Tans, P.; Andrews, A.; Kofler, J.; Helmig, D.; Guenther, D.; Dlugokencky, E.; Lang, P.; Newberger, T.; Wolter, S.; Hall, B.; Novelli, P.; Brewer, A.; Conley, S.; Hardesty, M.; Banta, R.; White, A.; Noone, D.; Wolfe, D.; Schnell, R., A new look at methane and nonmethane hydrocarbon emissions from oil and natural gas operations in the Colorado Denver-Julesburg Basin. *Journal of Geophysical Research: Atmospheres* **2014,** *119*, (11), 6836-6852, doi:doi:10.1002/2013JD021272.
46. Karion, A.; Sweeney, C.; Pétron, G.; Frost, G.; Michael Hardesty, R.; Kofler, J.; Miller, B. R.; Newberger, T.; Wolter, S.; Banta, R.; Brewer, A.; Dlugokencky, E.; Lang, P.; Montzka, S. A.; Schnell, R.; Tans, P.; Trainer, M.; Zamora, R.; Conley, S., Methane emissions estimate from airborne measurements over a western United States natural gas field. *Geophysical Research Letters* **2013,** *40*, (16), 4393-4397, doi:doi:10.1002/grl.50811.
47. Zhang, Y.; Gautam, R.; Pandey, S.; Omara, M.; Maasakkers, J. D.; Sadavarte, P.; Lyon, D.; Nesser, H.; Sulprizio, M. P.; Varon, D. J.; Zhang, R.; Houweling, S.; Zavala-Araiza, D.; Alvarez, R. A.; Lorente, A.; Hamburg, S. P.; Aben, I.; Jacob, D. J., Quantifying methane emissions from the largest oil-producing basin in the United States from space. *Science Advances* **2020,** *6*, (17), doi:10.1126/sciadv.aaz5120.
48. Pospíšil, J.; Charvát, P.; Arsenyeva, O.; Klimeš, L.; Špiláček, M.; Klemeš, J. J., Energy demand of liquefaction and regasification of natural gas and the potential of LNG for operative thermal energy storage. *Renewable and Sustainable Energy Reviews* **2019,** *99*, 1-15, doi:https://doi.org/10.1016/j.rser.2018.09.027.
49. Abrahams, L. S.; Samaras, C.; Griffin, W. M.; Matthews, H. S., Life Cycle Greenhouse Gas Emissions From U.S. Liquefied Natural Gas Exports: Implications for End Uses. *Environmental science & technology* **2015,** *49*, (5), 3237-3245, doi:10.1021/es505617p.
50. Mallapragada, D. S.; Reyes-Bastida, E.; Roberto, F.; McElroy, E. M.; Veskovic, D.; Laurenzi, I. J., Life cycle greenhouse gas emissions and freshwater consumption of liquefied Marcellus shale gas used for international power generation. *Journal of Cleaner Production* **2018,** *205*, 672-680, doi:https://doi.org/10.1016/j.jclepro.2018.09.111.
51. Sharafian, A.; Blomerus, P.; Mérida, W., Natural gas as a ship fuel: Assessment of greenhouse gas and air pollutant reduction potential. *Energy Policy* **2019,** *131*, 332-346, doi:https://doi.org/10.1016/j.enpol.2019.05.015.
52. Balcombe, P.; Anderson, K. E.; Speirs, J.; Brandon, N.; Hawkes, A., The Natural Gas Supply Chain: The Importance of Methane and Carbon Dioxide Emissions. *ACS Sustainable Chemistry & Engineering* **2016,** *5*, (1), 3-20, doi:10.1021/acssuschemeng.6b00144.
53. thinkstep AG *Life Cycle GHG Emission Study on the Use of LNG as Marine Fuel*; thinkstep: 2019; p 154,
54. UBA *Wie klimafreundlich ist LNG?*; 2019,
55. Littlefield, J.; Roman-White, S.; Augustine, D.; Pegallapati, A.; Zaimes, G. G.; Rai, S.; Cooney, G.; Skone, T. J. *Life Cycle Analysis of Natural Gas Extraction and Power Generation*; National Energy Technology Laboratory (NETL): 2019, https://www.netl.doe.gov/energy-analysis/details?id=3198

56. EPA - United States Environmental Protection Agency *Inventory of U.S. Greenhouse Gas Emissions and Sinks - 1990-2017*; EPA 430-P-19-001; EPA: Washington DC, 2019,

57. Wachsmuth, J.; Oberle, S.; Zubair, A.; Köppel, W. *Wie klimafreundlich ist LNG? - Kurzstudie zur Bewertung der Vorkettenemissionen bei Nutzung von verflüssigtem Erdgas (LNG)*; 1862-4359; Umweltbundesamt: Dessau-Roßlau, 2019; p 32, https://www.umweltbundesamt.de/publikationen/wie-klimafreundlich-ist-lng
